# Natural resistance to Meningococcal Disease related to CFH loci: Meta-analysis of genome-wide association studies

**DOI:** 10.1038/srep35842

**Published:** 2016-11-02

**Authors:** Federico Martinón-Torres, Eileen Png, Chiea Chuen Khor, Sonia Davila, Victoria J. Wright, Kar Seng Sim, Ana Vega, Laura Fachal, David Inwald, Simon Nadel, Enitan D. Carrol, Nazareth Martinón-Torres, Sonia Marcos Alonso, Angel Carracedo, Elvira Morteruel, Julio López-Bayón, Andrés Concha Torre, Cristina Calvo Monge, Pilar Azcón González de Aguilar, Elisabeth Esteban Torné, María del Carmen Martínez-Padilla, José María Martinón-Sánchez, Michael Levin, Martin L. Hibberd, Antonio Salas, ESIGEM network, Alberto Gómez-Carballa, Miriam Cebey, Natalia García Sánchez, Irene Rivero Calle, Antonio Justicia Grande, Jacobo Pardo-Seco, Ruth Barral-Arca, Sara Pischedda, María-José Currás-Tuala, Carmen Rodriguez-Tenreiro, Lorenzo Redondo-Collazo, Fernanda Pardo Sánchez, Jesús de la Cruz Moreno, Mª. Leticia Millán Miralles, José Luis García Rodríguez, Susana Rey García, Ana Hurtado Doce, Ángela Ferrer Barba, Manuel Ortiz Pallares, Alfredo Reparaz Romero, Juan Ignacio Muñoz Bonet, Manuel Silveira Cancela, Eider Oñate Bergara, Amaya Bustinza Arriortua, María Luisa Navarro Gómez, Mario Sánchez Fernández, Xavier Allué Martínez, Álvaro Castellanos Ortega, Servando Pantoja Rosso, César Pérez Caballero Macarrón, Natalia Molini Menchón, Francisco Giménez Sánchez, Manuel González-Ripoll Garzón, María del Mar Ballesteros García, José Manuel Sánchez Granados, Olga Serrano Ayestarán, Roman Payo, Sonia Cañadas Palazón, Maria Cruz León León, Susana Reyes Dominguez, David Arjona Villanueba, J. Antonio Alonso Martín, Concepción Goñi Orayen, Enrique Bernaola Iturbe, María Teresa Alonso Salas, Isabel Quintela Fernández, ESPID meningococcal consortium – UK, Robert Booy, Lachlan J. M. Coin, Hariklia Eleftherohorinou, Saul Faust, Rachel Galassini, Parviz Habibi, Elene Haralambous, Simon Kroll, Paul Langford, Nazima Pathan, Andrew J. Pollard, EUCLIDS consortium members - Imperial College London (www.euclids-project.eu), Farhana Abdulla, Paul Agapow, Evangelos Bellos, Shea Hamilton, Jethro A. Herberg, Clive Hoggart, Myrsini Kaforou, Meg Mashbat, Sobia Mustafa, Vanessa Sancho-Shimizu

**Affiliations:** 1 Translational Pediatrics and Infectious Diseases, Hospital Clínico Universitario de Santiago, Santiago de Compostela, Spain, and GENVIP Research Group (www.genvip.org), Instituto de Investigación Sanitaria de Santiago, Galicia, Spain; 2 Infectious Diseases, Genome Institute of Singapore, Singapore; 3 Human Genetics, Genome Institute of Singapore, Singapore; 4 Section of Paediatrics, Division of Infectious Diseases, Department of Medicine, Imperial College London, UK; 5 Fundación Pública Galega de Medicina Xenómica, Servizo Galego de Saúde (SERGAS), Instituto de Investigaciones Sanitarias (IDIS), and Grupo de Medicina Xenómica, Centro de Investigación Biomédica en Red de Enfermedades Raras (CIBERER), Universidade de Santiago de Compostela (USC), Santiago de Compostela, Spain; 6 Institute of Infection and Global Health, University of Liverpool, Liverpool, UK; 7 Unidade de Xenética, Departamento de Anatomía Patolóxica e Ciencias Forenses, Instituto de Ciencias Forenses, Facultade de Medicina, Universidade de Santiago de Compostela, and GenPop Research Group, Instituto de Investigaciones Sanitarias (IDIS), Hospital Clínico Universitario de Santiago, Galicia, Spain; 8 Unidad de Cuidados Intensivos Pediátricos (UCIP), Hospital de Cruces, Bilbao, Spain; 9 Unidad de Cuidados Intensivos Pediátricos (UCIP), Hospital Universitario Central de Asturias, Oviedo, Asturias, Spain; 10 Servicio de Pediatría, Hospital de Donostia, San Sebastián, Spain; 11 Unidad de Cuidados Intensivos Pediátricos (UCIP), Hospital Virgen de las Nieves de Granada, Granada, Spain; 12 Unidad de Cuidados Intensivos Pediátricos, Hospital Sant Joan de Deu, Barcelona, Spain; 13 Unidad de Cuidados Intensivos Pediátricos, Complejo Hospitalario de Jaen, Spain; 14 Center of Excellence in Genomic Medicine Research, King Abdulaziaz University, Jeddah, Saudi Arabia; 15 Departamento de Pediatría, Complejo Hospitalario de Orense, Galicia, Spain; 16 Servicio de Pediatría, Complejo Hospitalario Universitario de A Coruña, Galicia, Spain; 17 Servicio de Pediatría, Complejo Hospitalario Universitario de Vigo, Galicia, Spain; 18 Sección Cuidados Intensivos y Urgencias Pediátricas, Hospital Clínico Universitario de Valencia, Valencia, Spain; 19 Servicio de pediatría, Hospital da Costa de Burela, Galicia, Spain; 20 Unidad de Cuidados Intensivos Pediátricos, Hospital Gregorio Marañón de Madrid, Madrid, Spain; 21 Servicio de Pediatría, Hospital Josep Trueta de Girona, Girona, Spain; 22 Servicio de Pediatría, Hospital Juan XXIII de Tarragona, Tarragona, Spain; 23 Unidad de Cuidados Intensivos Pediátricos, Hospital Marqués de Valdecillas de Santander, Santander, Spain; 24 Unidad de Cuidados Intensivos Pediátricos, Hospital Puerta del Mar de Cádiz, Cádiz, Spain.; 25 Servicio de Pediatría, Hospital Ramón y Cajal de Madrid, Madrid, Spain; 26 Servicio de Pediatría, Hospital Rey Don Jaime de Castellón, Castellón, Spain.; 27 Servicio de Pediatría, Hospital Torrecárdenas de Almería, Almería, Spain.; 28 Unidad de Cuidados Intensivos Pediátricos, Hospital Universitario de Albacete, Albacete, Spain; 29 Servicio de Pediatría, Hospital Universitario de Salamanca, Salamanca, Spain; 30 Servicio de Pediatría, Hospital Vall d´Hebrón de Barcelona, Barcelona, Spain; 31 Unidad de Cuidados Intensivos Pediátricos, Hospital Virgen de la Arrixaca de Murcia, Murcia, Spain; 32 Servicio de Pediatría, Hospital Virgen de la Salud de Toledo, Toledo, Spain; 33 Servicio de Pediatría, Hospital Virgen del Camino de Navarra, Navarra, Spain; 34 Servicio de Pediatría, Hospital Virgen del Rocío de Sevilla, Sevilla, Spain; 35 Servicio de Pediatría, Hospital Virxe da Xunqueira, Galicia, Spain; 36 National Centre for Immunisation Research and Surveillance, University of Sydney.; 37 Department of Genomics of Common Disease, Imperial College, London, United Kingdom.; 38 School of Public Health, Faculty of Medicine, Imperial College London, London, United Kingdom.; 39 Paediatric Immunology and Infectious Diseases, Faculty of Medicine, University of Southampton, United Kingdom.; 40 Department of Paediatrics, University of Cambridge, Cambridge, United Kingdom.; 41 Oxford Vaccine Group Department of Paediatrics, University of Oxford and the NIHR Oxford Biomedical Research Centre, Oxford, United Kingdom.; 42 Centre for Infections, Health Protection Agency, London, United Kingdom.; 43 Department of Epidemiology and Biostatistics, Imperial College London, London, United Kingdom.

## Abstract

Meningococcal disease (MD) remains an important infectious cause of life threatening infection in both industrialized and resource poor countries. Genetic factors influence both occurrence and severity of presentation, but the genes responsible are largely unknown. We performed a genome-wide association study (GWAS) examining 5,440,063 SNPs in 422 Spanish MD patients and 910 controls. We then performed a meta-analysis of the Spanish GWAS with GWAS data from the United Kingdom (combined cohorts: 897 cases and 5,613 controls; 4,898,259 SNPs). The meta-analysis identified strong evidence of association (*P*-value ≤ 5 × 10^−8^) in 20 variants located at the *CFH* gene. SNP rs193053835 showed the most significant protective effect (Odds Ratio (OR) = 0.62, 95% confidence interval (C.I.) = 0.52–0.73; *P*-value = 9.62 × 10^−9^). Five other variants had been previously reported to be associated with susceptibility to MD, including the missense SNP rs1065489 (OR = 0.64, 95% C.I.) = 0.55–0.76, *P-value* = 3.25 × 10^−8^). Theoretical predictions point to a functional effect of rs1065489, which may be directly responsible for protection against MD. Our study confirms the association of *CFH* with susceptibility to MD and strengthens the importance of this link in understanding pathogenesis of the disease.

Meningococcal disease (MD), caused by *Neisseria meningitidis*, is an important cause of meningitis and septicaemia in children and young adults globally; it is associated with average mortality rates of 10% and long-term disability in survivors despite early diagnosis, antibiotic administration, and intensive care^1^,^2^. Global *N. meningitidis* serogroup distribution is varied and dynamic, making trends in disease epidemiology unpredictable^3^. Introduction of conjugate vaccines against serogroup C and A have drastically reduced its incidence in several countries^4^. However failure of wide implementation of the available conjugated vaccines against serogroups A, B, C, Y, and W-135 still makes MD a major challenge worldwide^5^.

There is now strong evidence that host genetic factors influence occurrence of MD, and a number of genes controlling susceptibility and severity of MD have been identified in candidate gene association studies^6^,^7^. Our previous genome-wide association study (GWAS) on a UK population sample, with validation in Western and southern European cohorts, identified genetic variations in the regulation of complement activation, complement factor H (*CFH*) and complement factor H related 3 (CFHR3) contributing to MD susceptibility^8^.

In order to identify new genetic associations with susceptibility to MD we carried out a GWAS in a cohort of Southern European (Spanish) MD cases and controls, and then undertook meta-analysis combining our South European GWAS with the previously published UK GWAS.

## Results and Discussion

Clinical and demographic data of Southern Europe (Spanish) and UK cohorts are summarized in Table 1.

In the Spanish MD cohort, we genotyped a total of 1,488 samples with 561,880 SNPs using the Illumina Quad 660W array, of which 42 samples had call rates <90% and were excluded. We detected 45 pairs of samples with suspected first-degree relationships. The sample with the lower call rate among each pair of relatives was removed to break any potential first-degree familial relationship in the cohort. In addition, a total of 69 samples were detected as significant ancestry outliers in the principal component analysis (PCA), and were also excluded, leaving a total of 422 MD cases and 910 controls for further imputation and association analyses. We also filtered the genotyped SNPs, removing those with call rate <95% (*n* = 1,258), those where the Hardy-Weinberg equilibrium (HWE) test showed significant deviation at *P*-value < 10^−7^ in controls (*n* = 12,754 SNPs), as well as SNPs not in human genome build 37 (*n* = 1,475). The final post QC dataset containing 546,393 SNPs was used to impute genotypes based on the 1000 Genomes Project Phase 1 reference data^9^ for an additional 6,749,004 SNPs, with impute probability of 90% and filtered with information score ≥0.90. We filtered the post-imputed dataset with the same QC criteria, including a minor allele frequency (MAF) filter of ≥1%, to have 5,440,063 SNPs in 422 MD cases and 910 controls. A mild degree of genetic stratification was observed (λGC value = 1.133) when we performed direct single SNP association analyses on the 5,440,063 SNPs, and hence we adjusted for the top 4 genetic axes of population ancestry on the association analysis, which lowered the λGC value to 1.03, Supplementary Data Figures S1A and S2A.

Details on, and data analysis for the UK GWAS cohort have been previously described^8^. From a starting number of 547 initially genotyped UK MD cases, a total of 72 samples were excluded from the study because they either failed genotyping (*n* = 6), were discrepant between clinically assigned and genotyped gender (*n* = 4), had a genotyping call rate below 95% (*n* = 17), were duplicates (*n* = 2), or were identified as a population outlier by PCA (*n* = 43). This left 475 UK MD cases for genetic association analysis. We used genome-wide genotyping data from UK healthy controls: 2,482 members of the 1958 Birth cohort (58BC), and 2,587 healthy blood donors from the National Blood Service (NBS) from the WTCCC2 project (http://www.wtccc.org.uk/ccc2). Out of 5069 controls, 4,703 passed stringent quality control filters and were included in final analysis. For this GWAS, a total of 516,963 SNPs, in common between the cases and controls, passed stringent quality control filters (per-SNP call rate of >95%, with HWE *P* > 10^−7^, present in human genome build 37). These SNPs were used to impute genotypes based on the 1000 Genomes Project Phase 1 reference data^9^, for an additional 7,453,462 SNPs; with impute probability of 90% and filtered with information score ≥0.90. We also filter the post imputed dataset with the same QC criteria, including a MAF filter of ≥1%, to have 5,365,603 SNPs in 475 UK MD cases and 4703 UK population controls.

Ancestry analysis of the UK sample collection showed that the MD cases and controls were well matched (Supplementary Data Figures S1B and S2B). In this light, the 1-degree of freedom (d.f.) score test for association performed in the UK dataset was not further adjusted for axes of genetic ancestry.

Given that this study has a total of 897 cases and 5614 controls, it has 0.969 power to reject the null hypothesis, considering that the risk allele frequency is at least 10%, the effect size (OR) is 0.6, and an uncorrected alpha threshold *P*-value = 0.05.

We observed minimal genome-wide inflation of the association test statistics when the UK and Spanish MD collections were analyzed separately (Supplementary Data Figure S1A,B), and thus formal meta-analysis for both sample collections was conducted under the fixed effects model using inverse-variance pooling as well as the random effects model, that were compared to show similar significance in *P*-values.Single SNP association tests from the meta-analysis between the Spanish and UK collections revealed an excess of significant *P*-values at the tail end of the distribution (Supplementary Data Figure S1C). Observed against a background of minimal genomic inflation, this suggests that at least some of these significant *P*-values (< 10^−5^) could represent true associations with MD.

**Figure 1:**
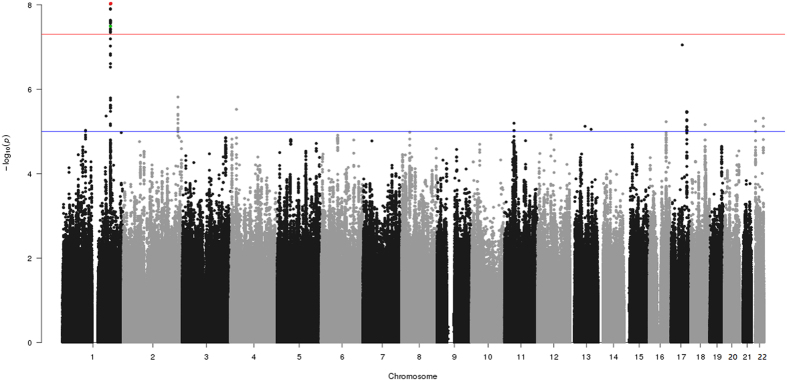
Genome-wide association plot for the Meningococcal meta-analysis between the Spanish and UK MD collections. The Y-axis denotes the strength of the association (−log10 *P*-value) for each SNP marker. The X-axis denotes individual chromosomes. The horizontal lines denote significant (*P*-value = 5 × 10^−8^) and suggestive (*P*-value = 10^−5^) evidence of association with disease. The red dot is the top SNP (rs193053835) with the minimal *P*-value, and the green dot is the functional candidate SNP (rs1065489).

A total of 20 SNPs surpassed genome-wide significance (*P*-value ≤ 5 × 10^−8^) in the meta-analysis study of the two European cohorts that jointly include 897 MD cases and 5,613 controls (Fig. 1; and Supplementary Data Figure S3A,B for Manhattan plots on individual cohorts). A heterogeneity test showed non-significant values, indicating that the association was in the same direction in the two cohorts. The 20 SNPs fall in the *CFH* gene (located on chromosome 1q32–q32.1) or very close to this gene (rs72482675).

**Figure 2:**
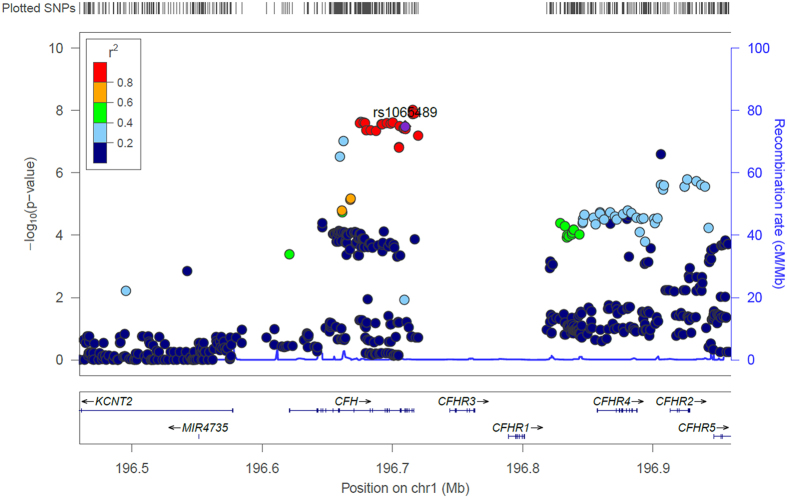
SNPs in the CFHR genes region were plotted according to its location in the genome (X-axis) against the combined meta-analysis −log10 *P*-value in the Y-axis. The candidate SNP; rs1065489 is denoted as the purple circle, whereas red circles represent SNPs in LD (r^2^ ≥ 0.8) with rs1065489. Recombination rates and LD values were plotted based on the 1000 Genomes European 2012 reference, in genome build hg19. See also Supplementary Data Figures S8 that covers the *CFHR3* and *CFHR1* regions using a call rate filter >80%.

We found the strongest evidence of association with protection to MD susceptibility at SNP rs193053835 (*P-value* = 9.62 × 10^−9^ per-allele OR = 0.62, 95% C.I. = 0.52–0.73) (Fig. 2) followed by rs72482675 (*P*-value = 1.25 × 10^−8^ per-allele OR = 0.63, 95% C.I. = 0.54–0.74) and rs105980 (*P*-value = 1.28 × 10^−8^ per-allele OR = 0.62, C.I. = 0.52–0.73); Table 2 and Supplementary Dataset S1. Other SNPs with *P*-values < 1 × 10^−5^ are shown in Supplementary Dataset S2. For the purpose of discovery novel candidate loci, we re-scanned the genome after conditioning on the top SNPs (rs1065489 and rs193053835; see Supplementary Data Figure S4), but we did not find other SNPs significantly associated (*P*-value ≤ 5 × 10^−8^) with MD.

We performed a stepwise conditional analysis to assess for the presence of independent signals among the 20 candidate SNPs. After conditioning for the top SNPs, the significant *P*-values observed in other SNPs disappeared (Supplementary Dataset S3). In addition, many of the SNPs were found to have collinearity, indicating that the 20 SNPs are strongly correlated with each other.

Five out of the 20 *CFH* associated variants were also reported to be associated with MD in Davila *et al.*^8^, namely, rs742855, rs1065489, rs11582939, rs11799595, and rs10489456. In addition, the SNP rs1065489 was additionally replicated in a Central European cohort^10^. We did not find an association with rs3753394; a SNP located in the promoter region that was previously reported to be associated with MD susceptibility by Haralambous *et al.*^11^. Consistently, all the studies point to a protective effect of these variants, with OR values ranging (in our study) from 0.62 to 0.72.

Although the SNP rs12085435, recently associated by Bradley *et al.*^12^ with invasive MD, was not genotyped in the present study, we did not find other SNPs associated within the *C8B* region. In addition, other SNPs recently associated with Age-related Macular Degeneration (AMD) risk located within the CFH region^13^ did not appeared as associated in our meta-analysis. Moreover, we did not find genetic variation associated within *CFHR3*, as previously reported^8^.

A total of 18 out of the 20 candidate SNPs located within the *CFH* gene, fall in noncoding regions. The exceptions are rs3753396 (*P-value* = 2.49 × 10^−8^ per-allele OR = 0.64, 95% C.I. = 0.54–0.75), that represents a synonymous change (exon 13; Gln672Gln), and rs1065489 (*P-value* = 3.25 × 10^−8^ per-allele OR = 0.64, 95% C.I. = 0.55–0.76) that represents a missense variant (exon 18; Glu936Asp).

The G allele at rs3753396 reaches the highest frequency in East Asia (50%), but it appears at high frequencies also in Europe and America (~18%); in Africa, however, the G variant reaches only 3.7% of the population (Supplementary Data Table S1). This SNP was also reported to be associated with AMD in Ref. 14.

Similarly, the minor allele T at the rs1065489 locus has a high frequency in East Asia (49.7%), while it reaches moderate frequencies in America (19.1%) and Europe (18.3%), and has a low frequency in Africa (3.7%). Of the 20 candidate SNPs in *CFH*, the rs1065489, located in exon 19, is the only one that represents a nonsynonymous substitution; and it is therefore more likely to be potentially functional. The data indicate that carriers of allele T have a protective effect against MD, with an OR = 0.64 in the merged cohorts (Table 2 and Supplementary Dataset S1). As already noticed in Davila *et al.*^8^, the incidence of MD inversely parallels the MAF of this SNP. Thus, sub-Saharan African populations have the highest rates of disease but the lowest frequency of this polymorphism (Supplementary Data Figure S5 and Table S1). Supplementary Data Table S2 shows different functional predictions for this variant; while a number of the predictions indicate that this variant is functionally tolerated, Polyphen2 using the database HDIV (recommended when evaluating loci potentially involved in complex phenotypes) indicates that rs1065489 is possibly damaging. Most interesting, the DANN score of this SNP receives a value of 0.985 (ranging from 0 to 1). It has been reported that this score has the best sensitivity and specificity when compared to comparable scores (e.g. CADD and FATHMM). Moreover, it is worth mentioning that this variant is highly phylogenetically conserved in other mammals (Supplementary Data Figure S6). In addition, Bradley *et al.*^12^ recently described an association of this SNP with genetic susceptibility to MD. It is important to note that all the SNPs in the *CFH* region are in high linkage disequilibrium (Fig. 2 and Supplementary Data Figure S7), and therefore, there could be other causal SNPs (in high LD with rs1065489) conferring protection against MD not captured in the present GWAs.

The present study adds further support to the association in *CFH* to the susceptibility of MD previously reported by Davila *et al.*^8^. Different polymorphisms at the *CFH* gene were also found to be associated in a number of diseases, including Atypical Hemolytic Uremic Syndrome^15^, or AMD^16^. The *CFH* gene encodes for the protein complement factor H, which plays an essential role regulating part of the complement system during an immune response. The complement system involves a cascade of proteins that work together in order to opsonize or kill invading pathogens. According to Schneider *et al.*^17^ *N. meningitidis* exploits the natural regulators (such as FH) to scape from the complement system and therefore from host immune control. The involvement of *CFH* and *CFHR* with MD appears to be through their role in inhibition of complement activation. Complement mediated killing is a fundamental mechanism of immunity to meningococcal invasion. Meningococci express a surface factor H binding protein (fHbp), which binds the host plasma protein FH and possibly FH related proteins to the bacterial surface with high affinity. The bacteria thus appears to use a “Trojan horse” strategy to evade complement mediated killing, using the host CFH to inhibit complement activation on the bacterial surface.

The main limitation of the present study is that the study design lacks external replication stage. Further studies would therefore be needed in order to confirm the present findings. In addition, SNP rs3753396 was found to be protective for MD but it has been reported to be a risk factor in AMD. This interesting finding deserves also further investigation.

Summarizing, the fact that different population-based genome-wide studies show consistent statistical association of the same SNPs strengthens the evidence in favor of an association of the *CFH* gene in MD susceptibility. All of the significant SNPs were identified to reside within a single haplotype, indicating that the SNPs are in LD and correlated with each other. Further evidence from the stepwise conditional analysis demonstrated that the significant *P*-values seen in each SNPs are not independent after conditioning for the top SNPs. The risk alleles of the 20 genome wide significant SNPs have odds ratio that are similar within the range of 0.62–0.72, which are protective towards MD. We found that the non-synonymous variant rs1065489 may be directly responsible for a protective effect in MD. However, further studies are needed in order to better understand the molecular and functional mechanisms that relate *CFH* variants and MD and to identify causal variants within the *CFH* region. Our findings support complement factor H as an important target for future development of therapeutic and preventive strategies against MD.

## Material and Methods

### Study participants

Diagnosis of MD was made in patients presenting with a characteristic purpuric or petechial rash, and clinical evidence of meningitis and/or septicaemia^6^,^18^,^19^. Diagnosis was confirmed microbiologically by culture of *N. meningitidis* from blood or CSF, or by PCR for meningococcal DNA in blood. Individuals in whom microbiological studies were negative, but presented with a characteristic clinical picture of shock, purpuric rash or meningitis, were also included once other pathogenic bacteria or virus infection were ruled out^20^. Table 1 summarizes the clinical and demographic data of the Spanish and UK cohorts. All patients included in the present study were of European descent. The initial Southern European cohort consisted of 496 MD cases presenting between 2006–2009 to hospitals included in the Spanish MD research network ESIGEM (www.esigem.org) comprising 43 pediatric intensive care units coordinated from the Hospital Clínico Universitario of Santiago de Compostela (Galicia, Spain); for more information on these samples see Ref. 8. The initial UK cohort consisted of 547 patients enrolled at UK hospitals between 1995 and 20078. Clinical details on the UK cohort have been previously reported^21^,^22^,^23^.

Each MD case cohort was accompanied by geographically matched healthy controls. Thus, 992 unrelated healthy Spanish ethnically matched controls were recruited at the same time as the MD cases; a subset of this sample has been previously described^24^. UK controls for GWAS have already been described^8^; in brief, the genotypic data consisted of 4,703 individuals from the Wellcome Trust Case-Control Consortium 2 (WTCCC2)^8^.

The research was performed in accordance with the ICH Harmonized Tripartite Guidelines for Good Clinical Practice, the convention of the Council of Europe on Human Rights and Biomedicine and with the ethical principles laid down in the Declaration of Helsinki. The project was submitted and approved by the ethics committee of each participating site, namely, Ethics Committee of Galicia (Spain) and NRES Committee London (United Kingdon). Parents information sheets and informed consent forms were also submitted and approved. Informed consent (Informed assent when applicable) was obtained from parents/legal guardians of subjects (subjects when applicable) included in the study prior to any study procedure. The study was performed in compliance with National and European normative that is applicable for human research and data protection. Confidentiality of the identity of the participants was also safeguarded.

### Genotyping and data quality control

DNA was extracted from blood samples using established laboratory techniques. The MD cases were genotyped using the Illumina Human 660W Quad BeadChips for the Spanish collection, and the Illumina Human 610 K Quad BeadChips for the UK collection following manufacturer instructions.

The Spanish controls were genotyped using the Illumina 660W Quad BeadChip. The UK controls (drawn from the Wellcome Trust Case-Control Consortium 2; WTCCC2) were genotyped using the Illumina HumanHap 1.2M chip. Access to raw data for this control dataset was granted by The Wellcome Trust Consortium Data Access Committee for our study.

After pre-phasing the Spanish and UK GWAS datasets using SHAPEIT^25^, IMPUTE2 was used to perform imputation for additional SNPs genotype using the 1000 Genomes phase 1 (Feb 2012 release) cosmopolitan populations, as reference; Ref. 9. We used cosmopolitan multi-ethnic groups as reference for imputation because there is evidence performed on cosmopolitan panels *versus* population specific panels demonstrating that in general, the additional unrelated samples help to improve the imputation accuracy, in particular for the low frequency SNPs. Moreover, this procedure does not seem to penalize or reduce the accuracy, even when additional redundant samples are added as reference in the imputation^26^.

A selection of stringent quality control (QC) filters were applied to the genome-wide genotyping data to remove poorly performing SNPs and samples, using tools implemented in PLINK version 1.07 (Ref. 27). SNPs that had >5% of missing genotypes, gross departure from HWE (test for HWE showing significant deviation at *P* -value < 10^−7^ in controls) or were of MAF below 1% were excluded from downstream analysis. For QC, samples with an overall genotyping call rate of <95% were excluded from analysis. Most of the SNPs located in the *CFHR1* and *CFHR3* genes were filtered due to a poor call rate at this region. We therefore used a more relaxed filter (call rate >80%) in order to retain SNPs at these genes. However, the combined meta-analysis did not revealed better SNP candidates (Supplementary Data Figure S8A,B,C).

Samples were subjected to biological kinship verification by using the principle of variability in allele sharing according to the degree of relationship. Identity-by-state (IBS) information was derived using PLINK. Pairs of samples predicted to be first-degree relatives based on IBS sharing were identified, and the sample with the lower call rate of each pair was excluded from further analysis.

We combined the Spanish or UK samples with the Africans (YRI), East Asians (CHB, CHS, JPT), and samples with European descent (CEU, GBR, FIN, ISB, TSI) of the 1000 genomes project^28^,^29^, and used their LD-pruned SNPs to perform PCA as implemented in EIGENSTRAT^30^ and extracted the values of PC1 and PC2 to plot Supplementary Data Figure S2A,B. We also performed independent PCA on the Spanish GWAS dataset that consisted of 1332 post-QC samples, and extracted the top 4 genetic axes (PC1–PC4) of population ancestry to include as covariates in the SNP association analysis.

### Statistical analysis

A meta-analysis was carried out on the GWAS data from the Spanish and the UK cohort. Statistical analyses for association were undertaken using an additive model logistic regression as implemented in PLINK v1.07; Ref. 27. Principal component (PC) analysis as implemented in EIGENSTRAT^30^ was undertaken to account for spurious genetic associations resulting from ancestral differences of individual SNPs. PC plots were performed using the R statistical program package (www.r-project.org/).

For the independent GWAS data from Spain and UK, analysis of association with MD was undertaken using a 1 d.f. logistic regression test, which allows further adjustment of incorporating additional principal components as covariates of population stratification. The combined meta-analysis on the results of both Spanish and UK collections was performed using both the inverse-variance fixed effects method as well as the random effects model.

LocusZoom^31^ was used to plot the CFHRs genes regional association plot using the combined fixed effect meta-analysis P value of SNPs located in this region. LD in the plot was estimated based on the 1000 genomes EUR population and using the hg19 genome build coordinates.

R^2^ correlation values and the D′ CI method of Gabriel *et al.*^32^ as implemented in Haploview^33^ were used to plot the pairwise LD map between the 20 genome wide significant SNPs within the Spanish GWAS samples or the UK samples.

Functional annotation of gene variants was carried out using ANNOVAR^34^. ANNOVAR obtains from high-throughput sequencing data, different variant functional predictions, such as: (a) SIFT (Sorting Intolerant From Tolerant)^35^ that predicts whether an amino acid substitution is likely to affect protein function based on sequence homology and the physico-chemical similarity between the alternate amino acids, (b) PolyPhen2^36^: prediction of functional effects of human nsSNPs, (c) LRT (Likelihood Ratio Test)^37^ identifies a subset of deleterious mutations that disrupt highly conserved amino acids within protein-coding sequences, (d) MutationTaster^38^ evaluates the disease-causing potential of DNA sequence alterations, (e) MutationAssesor: predicts the functional impact of amino-acid substitutions in proteins, and (f) FATHMM or Functional Analysis Through Hidden Markov Models^39^. CADD (Combined Annotation Dependent Depletion)^40^ was also used for scoring the deleteriousness of single nucleotide variants. The algorithm that runs in the DANN score uses the exact same training and annotation data as CADD, but using a different machine learning approach^41^,^42^.

## Additional Information

**How to cite this article**: Martinón-Torres, F. *et al.* Natural resistance to Meningococcal Disease related to CFH loci: Meta-analysis of genome-wide association studies. *Sci. Rep.* **6**, 35842; doi: 10.1038/srep35842 (2016).

**Publisher’s note**: Springer Nature remains neutral with regard to jurisdictional claims in published maps and institutional affiliations.

## Supplementary Material

Supplementary Information

Supplementary Dataset S1

Supplementary Dataset S2

Supplementary Dataset S3

## Figures and Tables

**Table 1:** Demographic and clinical data of the cohorts successfully genotyped in the GWAS.

Characteristics	Spanish GWAS		UK GWAS	
	*n*_a_	*n*_b_	*n*_a_	*n*_b_
Number of male cases (%)	419	255 (60.8)	442	237 (53.6)
Cases of meningococcal meningitis (%)	397	56 (14.1)	439	59 (13.44)
Cases of meningococcal septicaemia (%)	397	185 (46.6)	439	280 (63.78)
Cases of meningococcal meningitis and septicaemia (%)	397	156 (39.3)	439	100 (22.78)
Median age at admission (IQR^b^)	419	2.3 (0.9–5.1)	442	3.32 (1.36–8.24)
Median GMSPS (IQR)	324	0 (0–2)	394	9 (6–12)
Inotropes (%)	381	183 (48.03)	441	268 (60.77)
Mechanical ventilation (%)^c^	381	183 (48.03)	442	321 (72.62)
Skin graft (%)	381	8 (2.09)	442	16 (3.62)
Amputation (%)	381	9 (2.36)	442	4 (0.90)
Death (%)	419	17 (4.05)	442	18 (4.07)
Controls (European ancestry)	992	992	4703	4703

*n*_a_: total number of informative cases/controls; *n*_*b*_: number of cases/controls carrying the phenotype/condition indicated in rows. Note that there were clinical data for 442 out of the 475 UK MD cases used in the present study.

IQR: interquartile range. *n*: total number of informative cases. GMSPS: Glasgow meningococcal prognostic score.

In the Spanish cohort, mechanical ventilation refers to any kind of respiratory support, including noninvasive ventilation.

**Table 2:** SNPs located at the *CFH* gene (chromosome 1) statistically associated with MD in the merged ESIGEM-UK cohort.

SNP	Position (BP)	Gene location	A1	Fixed-*P*	Fixed-OR	95% CI	Random-*P*	Random-OR	95% CI	Q	I^2^	Spanish IS	UK IS
rs193053835	196715667	Intron	T	9.62 × 10^−09^	0.62	0.52–0.73	9.62 × 10^−09^	0.62	0.52–0.73	0.78	0	0.962	0.965
rs72482675	196716924	500 bp Downstream	G	1.25 × 10^−08^	0.63	0.54–0.74	1.25 × 10^−08^	0.63	0.54–0.74	0.82	0	0.983	0.989
rs105980	196715666	Intron	A	1.28 × 10^−08^	0.62	0.52–0.73	1.28 × 10^−08^	0.62	0.52–0.73	0.83	0	0.962	0.965
rs6695321	196675861	Intron	G	2.33 × 10^−08^	0.72	0.64–0.81	3.53 × 10^−07^	0.72	0.63–0.82	0.27	16.61	0.997	0.998
rs12406047	196677898	Intron	T	2.38 × 10^−08^	0.64	0.54–0.75	2.38 × 10^−08^	0.64	0.54–0.75	0.77	0	0.987	0.989
rs11799595*	196700322	Intron	C	2.38 × 10^−08^	0.64	0.55–0.75	2.38 × 10^−08^	0.64	0.55–0.75	0.62	0	0.992	0.995
rs3753396	196695742	Synonymous	G	2.49 × 10^−08^	0.64	0.54–0.75	2.49 × 10^−08^	0.64	0.54–0.75	0.7	0	0.992	0.995
rs1048663	196674982	Intron	A	2.52 × 10^−08^	0.64	0.54–0.75	2.52 × 10^−08^	0.64	0.54–0.75	0.69	0	0.986	0.989
rs74213209	196679010	Intron	G	2.55 × 10^−08^	0.64	0.54–0.75	2.55 × 10^−08^	0.64	0.54–0.75	0.69	0	0.986	0.989
rs10922107	196698651	Intron	T	2.61 × 10^−08^	0.64	0.55–0.75	2.61 × 10^−08^	0.64	0.55–0.75	0.62	0	0.992	0.995
rs12402808	196691625	Intron	A	2.71 × 10^−08^	0.64	0.55–0.75	2.71 × 10^−08^	0.64	0.55–0.75	0.62	0	0.992	0.995
rs11801630	196692148	Intron	T	2.71 × 10^−08^	0.64	0.55–0.75	2.71 × 10^−08^	0.64	0.55–0.75	0.62	0	0.992	0.995
rs742855*	196705520	Intron	C	3.15 × 10^−08^	0.64	0.55–0.76	3.15 × 10^−08^	0.64	0.55–0.76	0.66	0	0.999	0.997
rs1065489*	196709774	Missense	T	3.25 × 10^−08^	0.64	0.55–0.76	3.25 × 10^−08^	0.64	0.55–0.76	0.84	0	—	—
rs11799380	196708455	Intron	G	3.77 × 10^−08^	0.65	0.55–0.76	3.77 × 10^−08^	0.65	0.55–0.76	0.8	0	0.999	0.999
rs2336221	196708891	Intron	T	3.77 × 10^−08^	0.65	0.55–0.76	3.77 × 10^−08^	0.65	0.55–0.76	0.8	0	1	0.999
rs11582939*	196710157	Intron	A	3.87 × 10^−08^	0.65	0.55–0.76	3.87 × 10^−08^	0.65	0.55–0.76	0.8	0	—	—
rs1831280	196683274	Intron	G	4.22 × 10^−08^	0.64	0.55–0.76	4.22 × 10^−08^	0.64	0.55–0.76	0.8	0	0.986	0.989
rs11584505	196679927	Intron	C	4.35 × 10^−08^	0.65	0.55–0.76	4.35 × 10^−08^	0.65	0.55–0.76	0.79	0	0.986	0.989
rs10489456*	196687515	Intron	A	4.56 × 10^−08^	0.65	0.55–0.76	4.56 × 10^−08^	0.65	0.55–0.76	0.73	0	0.992	0.995

An asterisk indicates those SNPs that were reported previously as associated with MD susceptibility. Position in base pairs (bp) are given according to GRCh37/hg19.

A1: risk allele; Fixed-*P*: fixed-effects meta-analysis *P*-value; Fixed.OR: fixed-effects meta-analysis odds ratio estimate with reference to the risk allele; in brackets is the 95% confidence interval; Random-*P*: random-effects meta-analysis *P*-value; Random-OR: Random-effects meta-analysis odds ratio estimate with reference to the risk allele; in brackets is the 95% confidence interval. Q: *P*-value for Cochrane’s Q statistic; I^2^: I-squared index between sample collections, with values ranging from 0 (no heterogeneity) to 100 (very high heterogeneity). I^2^ value < 50 indicates heterogeneity is not significant. Spanish IS: information on info score for SNPs that were imputed in the Spanish GWAS cohort. UK IS: information score for SNPs that were imputed in the UK GWAS cohort. rs1065489 and rs11582939 were directly genotyped.
