# Supplementary material for: Natural resistance to Meningococcal Disease related to CFH loci: Meta-analysis of genome-wide association studies
Source: Sci Rep. 2016 Nov 2;6:35842. doi: 10.1038/srep35842 (PMC5090968; doi:10.1038/srep35842)
Supplement: Supplementary Information [file srep35842-s1.pdf]

SUPPLEMENTARY DATA

Natural resistance to Meningococcal Disease related to CFH loci: Meta-analysis of genome-wide association studies

Federico Martinón-Torres, Eileen Png, Chiea Chuen Khor, Sonia Davila, Victoria J. Wright, Kar Seng Sim, Ana Vega, Laura Fachal, David Inwald, Simon Nadel, Enitan D Carrol, Nazareth Martinón-Torres, Sonia Marcos Alonso, Angel Carracedo, Elvira Morteruel, Julio López-Bayón, Andrés Concha Torre, Cristina Calvo Monge, Pilar Azcón González de Aguilar, Elisabeth Esteban Torné, María del Carmen Martínez-Padilla, José María Martinón-Sánchez, Michael Levin, Martin L. Hibberd, Antonio Salas, ESIGEM network, ESPID meningococcal consortium, EUCLIDS consortium

Contents

Figure S1. Quantile-quantile plot showing the observed and expected distribution of the association *P*-values (expressed as a 1 d.f. score test on logistic regression) on the MD cases from Spain and the Spanish controls (λ_{GC} =1.03) (**A**), the MD cases from UK and the WTCCC2 controls (λ_{GC} =1.02) (**B**). And the combined meta-analysed results of both Spanish and UK MD GWASs (λ_{GC} = 1.03) (**C**).

Figure S2. Analysis of genetic ancestry of the MD cases from Spain and the Spanish controls (ESIGEM) (**A**) and the MD cases from UK and the WTCCC2 controls (UKMGC) (**B**), with 1000 genomes populations from Africa (YRI), East Asia (CHB, CHS, JPT), and Europeans (CEU, FIN, GBR, IBS, TSI). The top two principal components of genetic ancestry (PC1 on the X-axis and PC2 on the Y-axis) are shown.

Figure S3. Genome-wide association plot for the Spanish Meningococcal GWAS (A) and the UK Meningococcal GWAS (B). The Y-axis denotes the strength of the association (-log10 *P*-value) for each SNP marker. The X-axis denotes individual chromosomes. The horizontal lines denote significant (*P*-

value = 5 x 10^{-8}) and suggestive (*P*-value = 10^{-5}) evidence of association with disease.

Figure S4. Genome-wide association plot for the Meningococcal meta-analysis after conditioning for the top SNP rs193053835. The Y-axis denotes the strength of the association (-log10 *P*-value) for each SNP marker. The X-axis denotes individual chromosomes. The horizontal lines denote significant (*P*-value = 5 x 10^{-8}) and suggestive (*P*-value = 10^{-5}) evidence of association with disease.

Figure S5. Pie charts of population allele frequencies at rs1065489 in 1000 Project Phase 3 (ENSEMBL).

Figure S6. Phylogenetic context of rs1065489, as inferred from ENSEMBL as observed in six eutherian mammals.

Figure S7. Pairwise LD defined by the D′ CI method between the 20 genome wide significant SNPs within (**A**) the Spanish or (**B**) the UK GWAS samples, and using R^2 within (**C**) the Spanish and (**D**) the UK GWAS samples.

Figure S8. SNPs in the CFHR genes region from the Spanish (**A**), the UK (**B**) and the meta-analysis data (**C**) were plotted according to its location in the genome (X-axis) against the combined meta-analysis –log10 *P*-value in the Y-axis and using a call rate filter > 80%. The candidate SNP; rs1065489 is denoted as the purple circle, whereas red circles represent SNPs in LD ($r^2 \geq 0.8$) with rs1065489. Recombination rates and LD values were plotted based on the 1000 Genomes European 2012 reference, in genome build hg19.

Table S1. Population parameters of the candidate SNPs observed in the meta-analysis carried out in the present study using ENGINES[42].

Table S2. Pathogenicity and deleteriousness scores at rs1065489.

Figure S1A

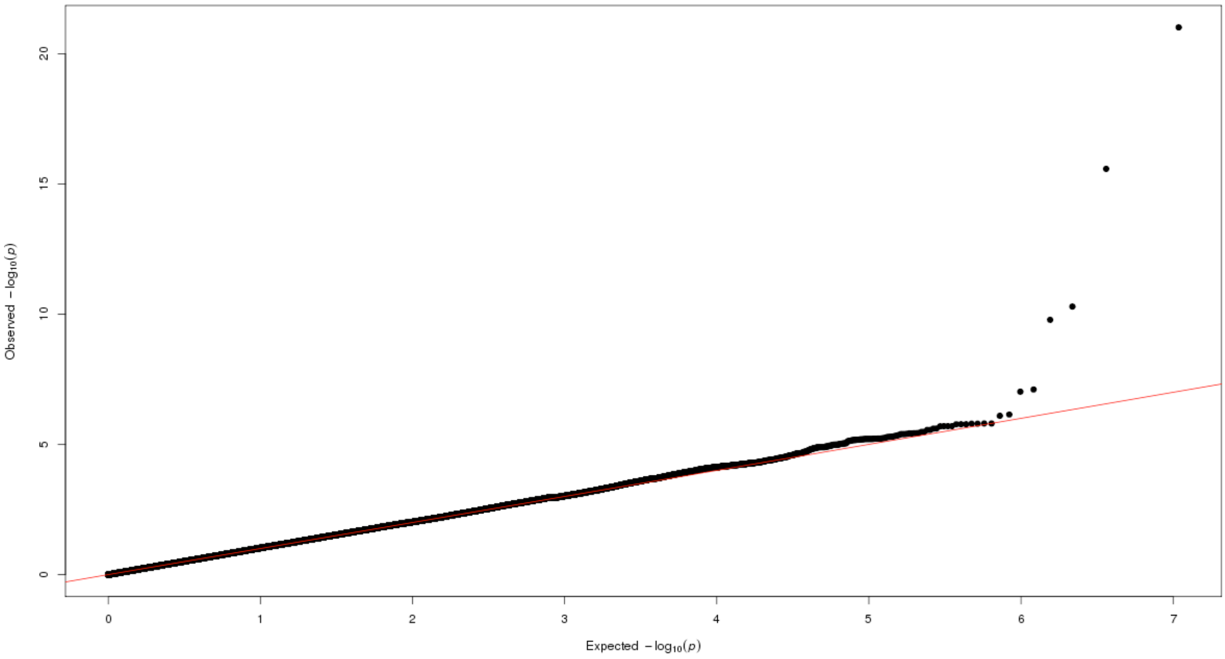

Figure S1B

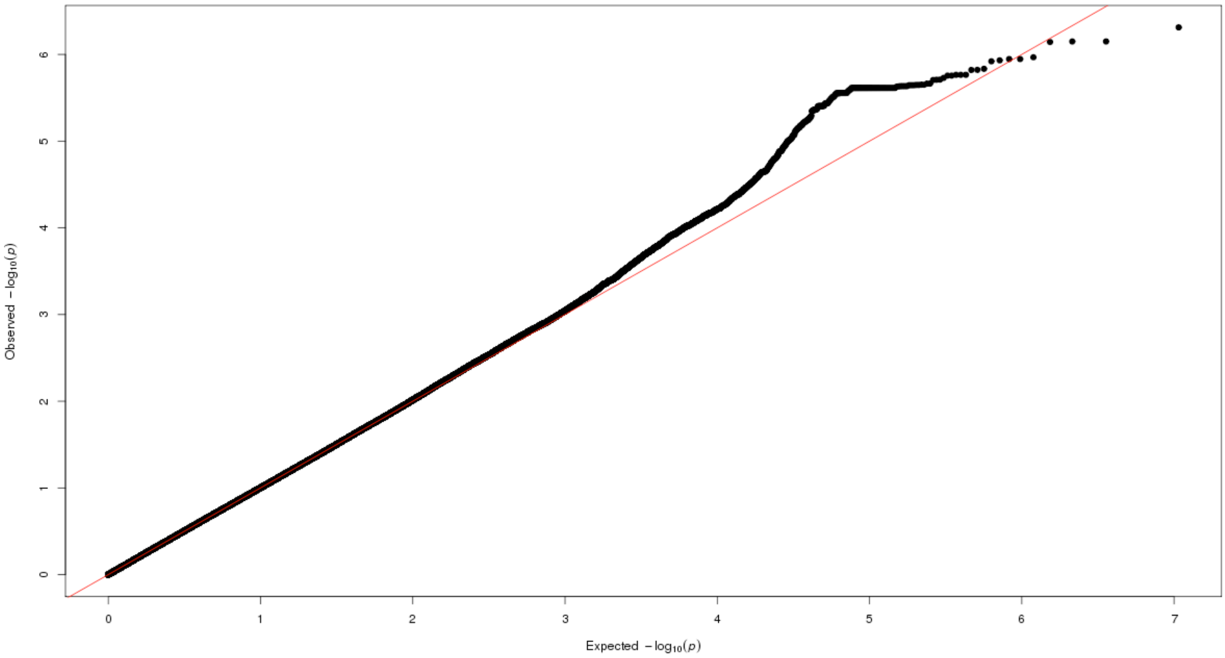

Figure S1C

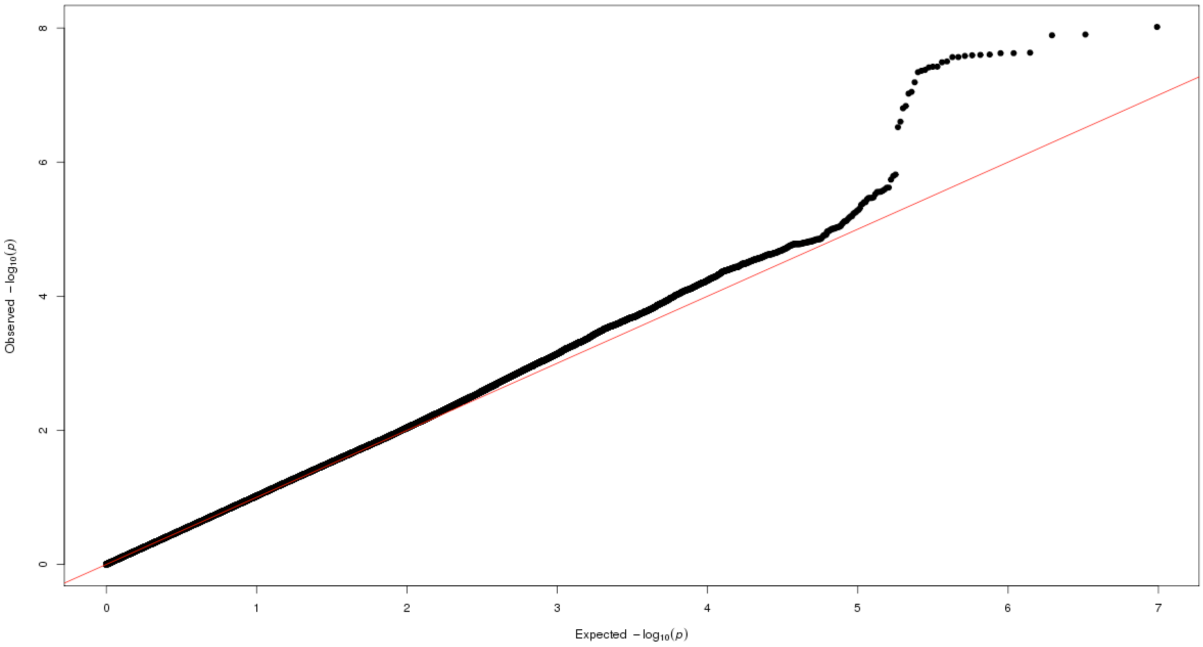

Figure S2A

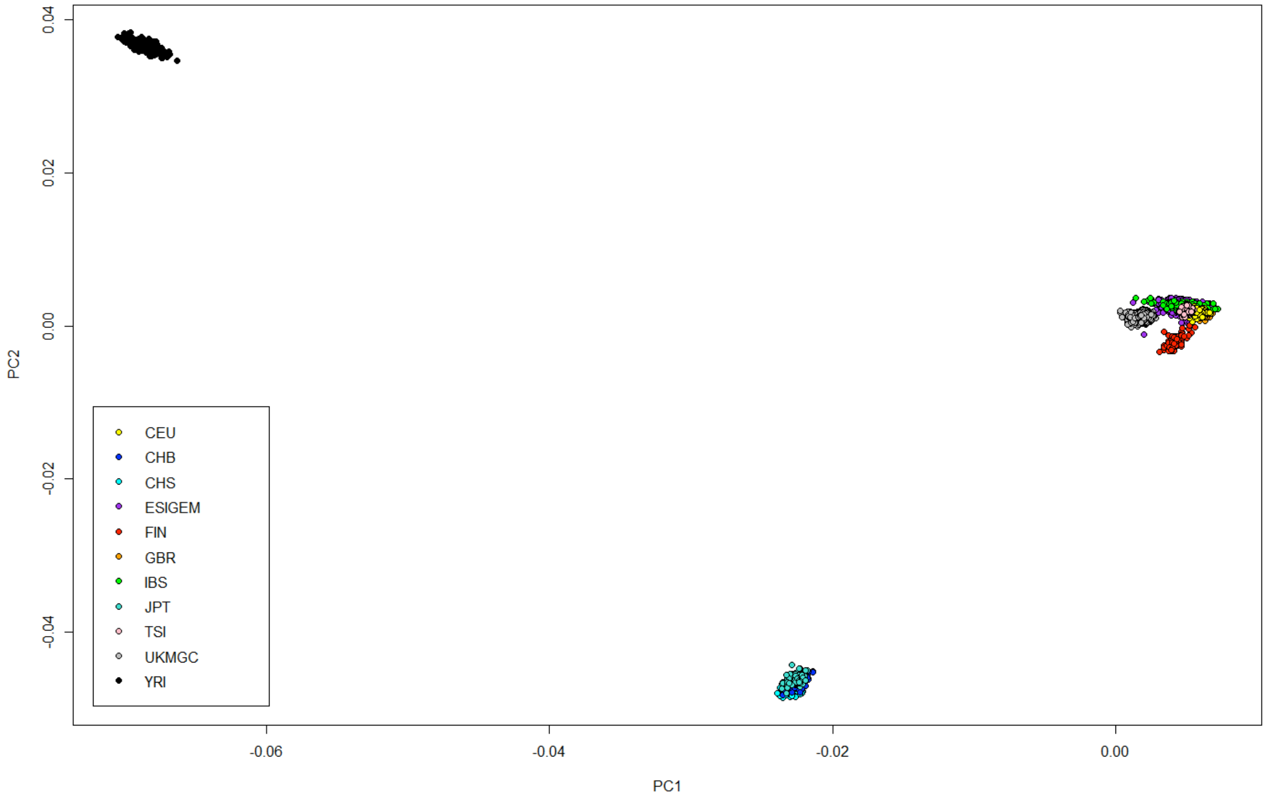
0.04
0.02
0.00
-0.02
-0.04
PC2
CEU
CHB
CHS
ESIGEM
FIN
GBR
IBS
JPT
TSI
UKMGC
YRI
-0.06
-0.04
-0.02
0.00
PC1

Figure S2B

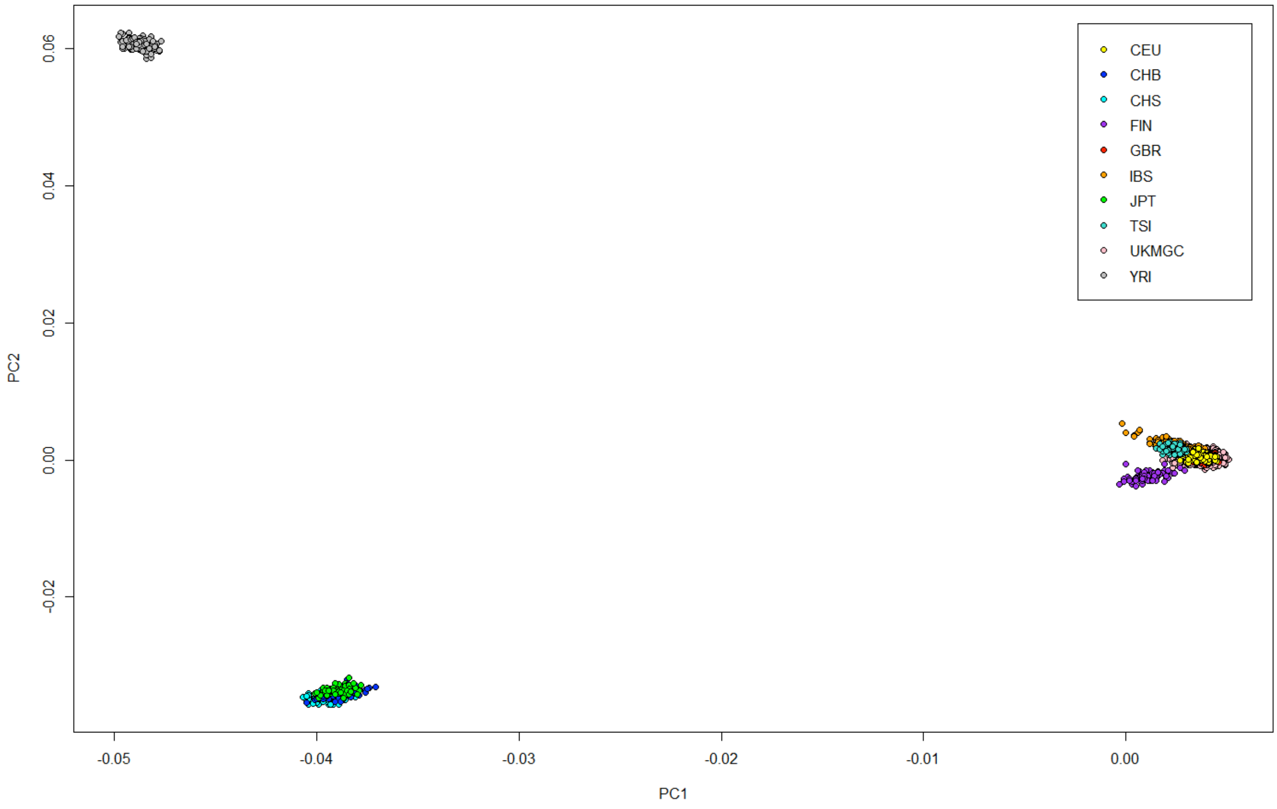
CEU
CHB
CHS
FIN
GBR
IBS
JPT
TSI
UKMGC
YRI
PC2
0.06
0.04
0.02
0.00
-0.02
-0.05
-0.04
-0.03
-0.02
-0.01
0.00
PC1

Figure S3A

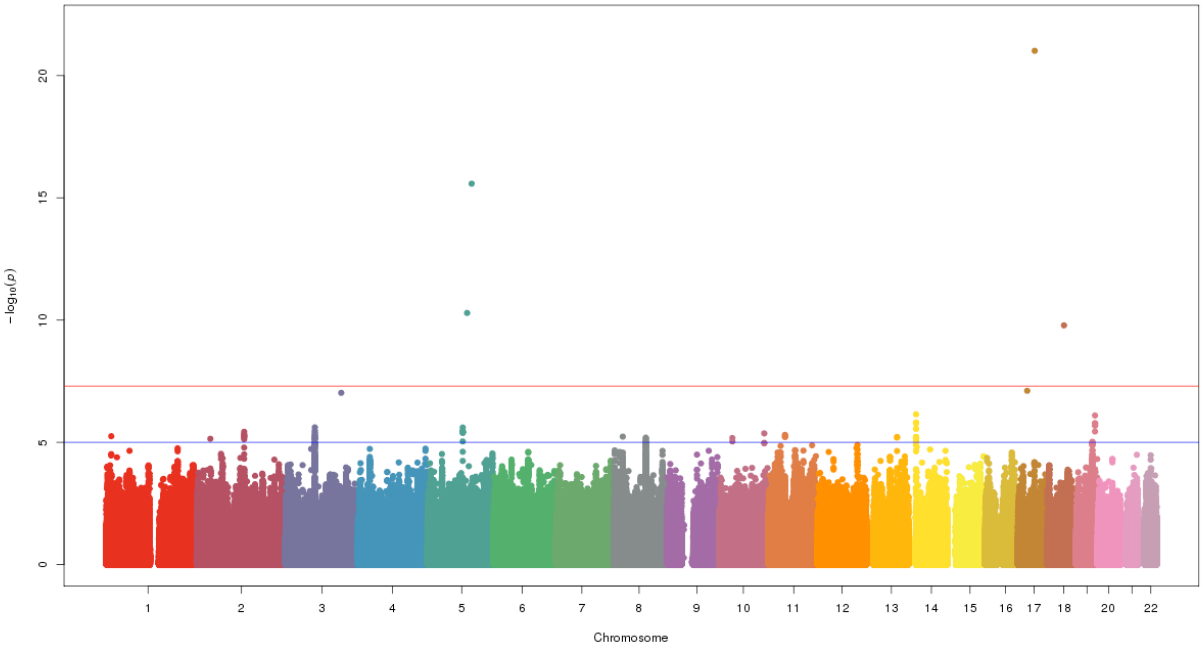

$-\log_{10}(p)$
0
5
10
15
20
1
2
3
4
5
6
7
8
9
10
11
12
13
14
15
16
17
18
20
22
Chromosome

Figure S3B

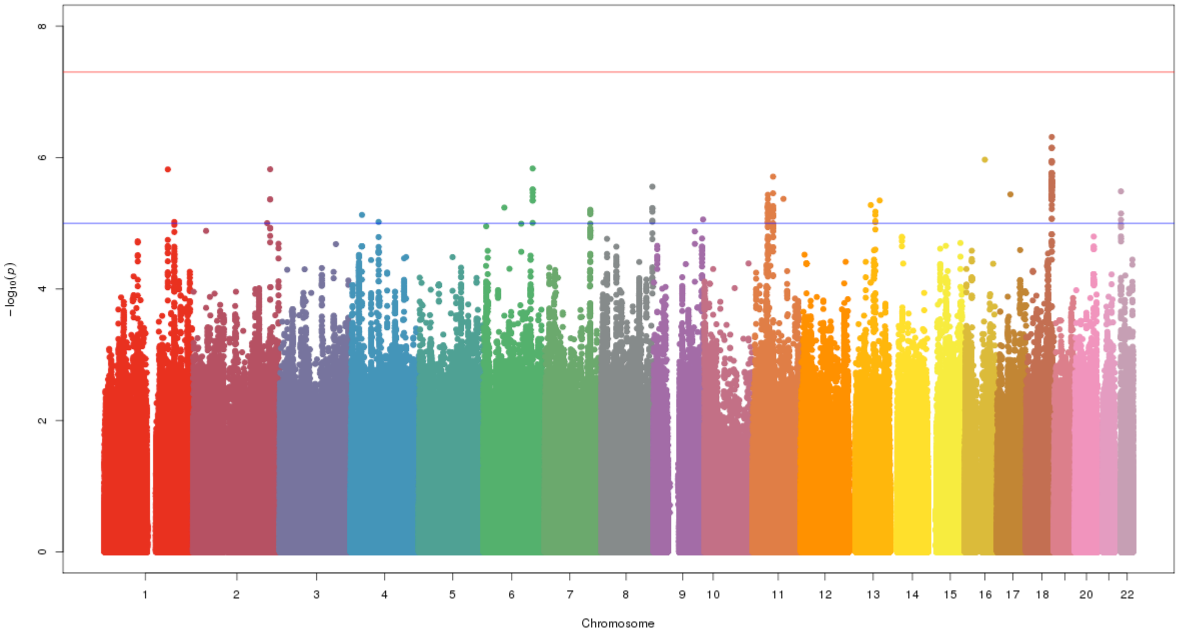

8
6
4
2
0
$-\log_{10}(p)$
1
2
3
4
5
6
7
8
9
10
11
12
13
14
15
16
17
18
20
22
Chromosome

Figure S4

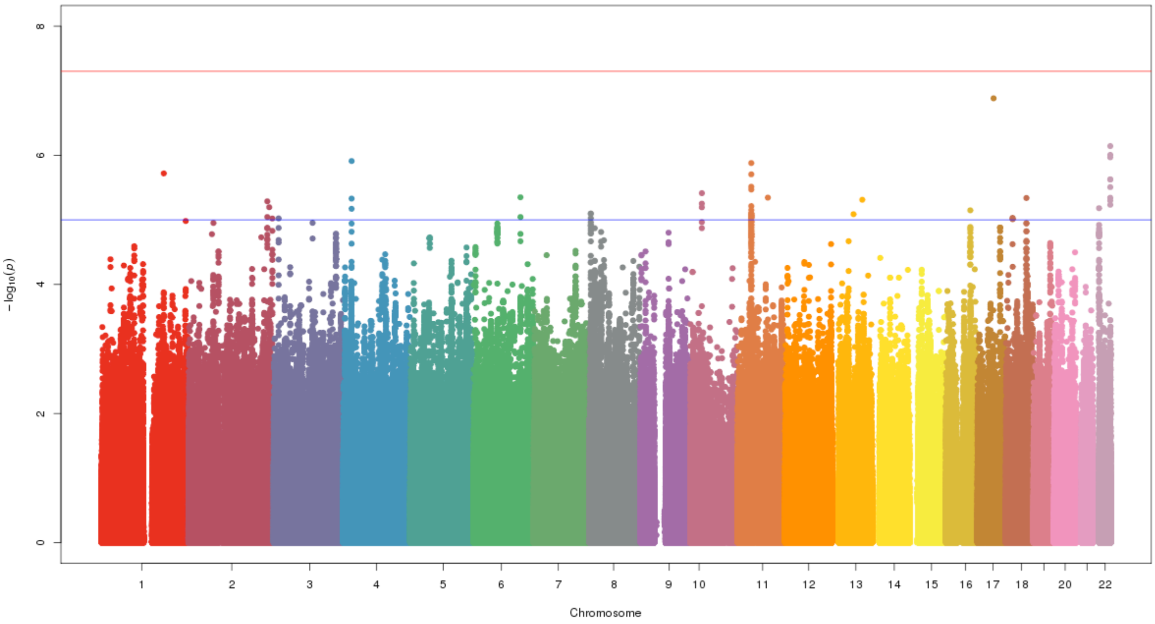

−log10(p)
0
2
4
6
8
1
2
3
4
5
6
7
8
9
10
11
12
13
14
15
16
17
18
20
22
Chromosome

Figure S5

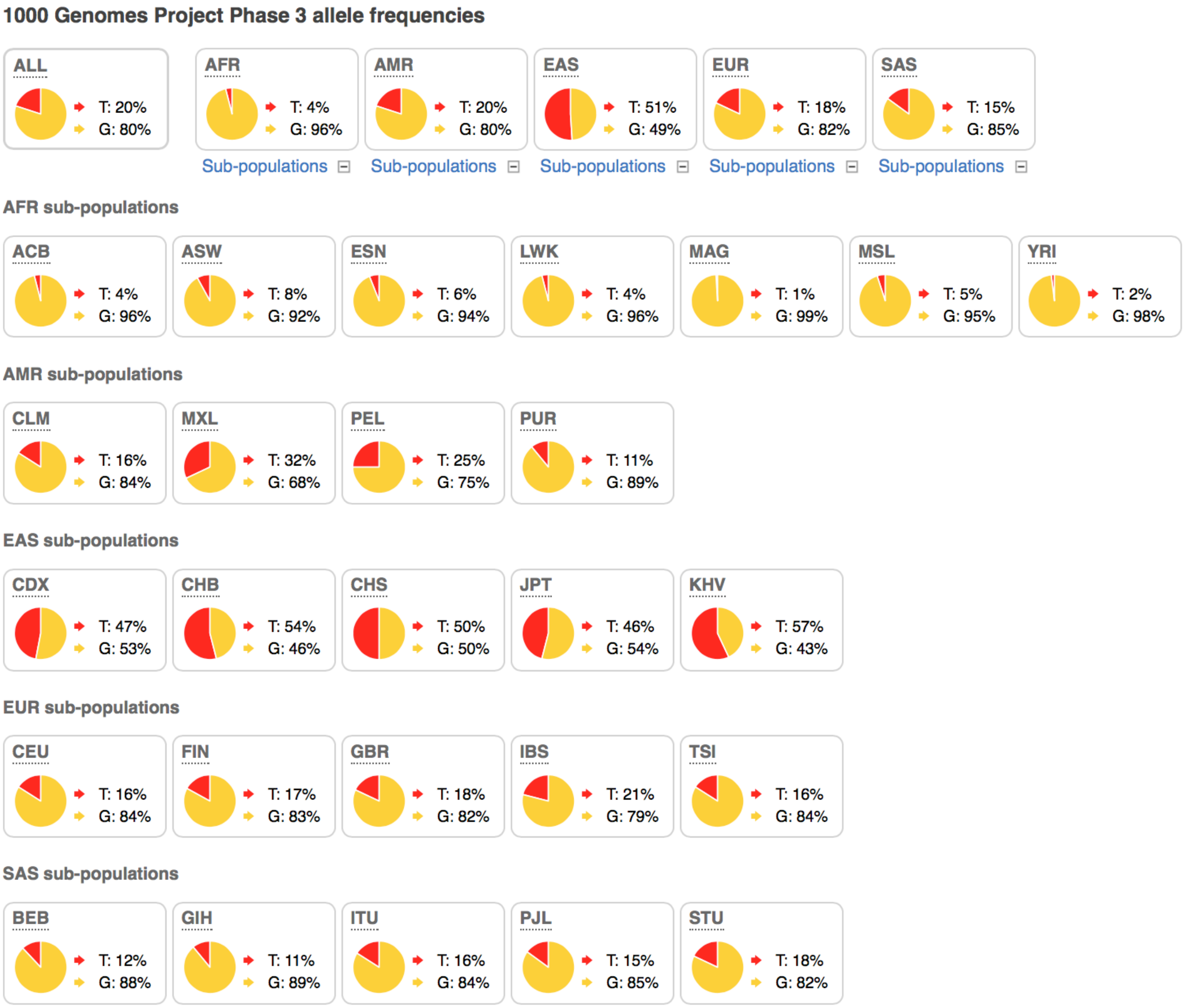
1000 Genomes Project Phase 3 allele frequencies
ALL T: 20% G: 80%
AFR T: 4% G: 96%
AMR T: 20% G: 80%
EAS T: 51% G: 49%
EUR T: 18% G: 82%
SAS T: 15% G: 85%
Sub-populations
Sub-populations
Sub-populations
Sub-populations
Sub-populations
AFR sub-populations
ACB T: 4% G: 96%
ASW T: 8% G: 92%
ESN T: 6% G: 94%
LWK T: 4% G: 96%
MAG T: 1% G: 99%
MSL T: 5% G: 95%
YRI T: 2% G: 98%
AMR sub-populations
CLM T: 16% G: 84%
MXL T: 32% G: 68%
PEL T: 25% G: 75%
PUR T: 11% G: 89%
EAS sub-populations
CDX T: 47% G: 53%
CHB T: 54% G: 46%
CHS T: 50% G: 50%
JPT T: 46% G: 54%
KHV T: 57% G: 43%
EUR sub-populations
CEU T: 16% G: 84%
FIN T: 17% G: 83%
GBR T: 18% G: 82%
IBS T: 21% G: 79%
TSI T: 16% G: 84%
SAS sub-populations
BEB T: 12% G: 88%
GIH T: 11% G: 89%
ITU T: 16% G: 84%
PJL T: 15% G: 85%
STU T: 18% G: 82%

Figure S6

**Human ›**	chromosome:GRCh38:1:196740634:196740654:1
**Chimpanzee ›**	chromosome:CHIMP2.1.4:1:175454801:175454821:1
**Gorilla ›**	chromosome:gorGor3.1:1:176494878:176494898:1
**Vervet-AGM ›**	chromosome:ChlSab1.1:25:32577812:32577832:-1
**Olive baboon ›**	chromosome:PapAnu2.0:1:167150656:167150676:-1
**Rabbit ›**	chromosome:OryCun2.0:16:74300301:74300321:-1

```
                         Y    K      M
Human           CTCCACCTGAGATTTCTCATG
Chimpanzee      CTCCACCTGAGATTTCTCATG
Gorilla         CTCCACCTGAGATTTCTCATG
Vervet-AGM      CTCCACCTGAGATTTCTCATG
Olive baboon    CTCCACCTGAGATTTCTCATG
Rabbit          CTCCACCTGAGATTTCTAATG
```

Figure S7A

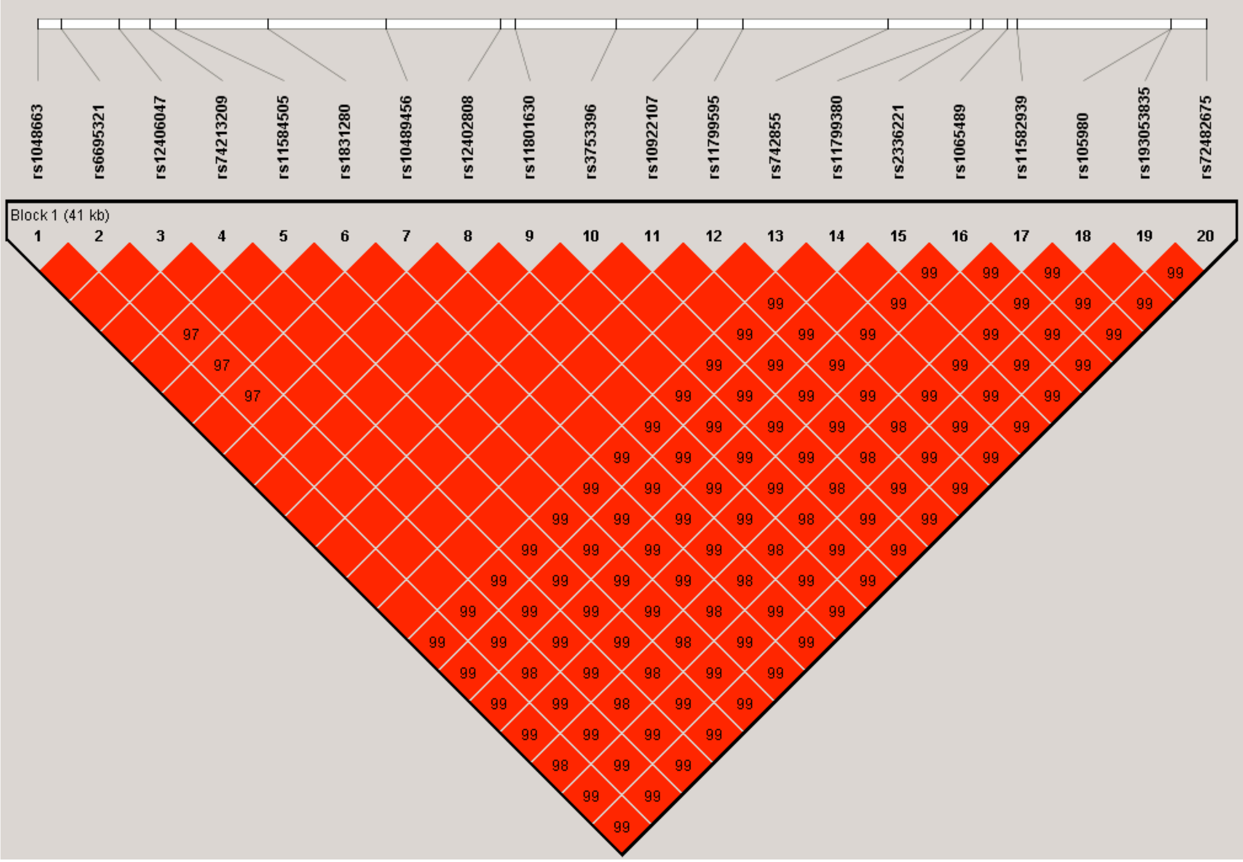
rs1048663
rs6695321
rs12406047
rs74213209
rs11584505
rs1831280
rs10489456
rs12402808
rs11801630
rs3753396
rs10922107
rs11799595
rs742855
rs11799380
rs2336221
rs1065489
rs11582939
rs105980
rs193053835
rs72482675
Block 1 (41 kb)
1 2 3 4 5 6 7 8 9 10 11 12 13 14 15 16 17 18 19 20

Figure S7B

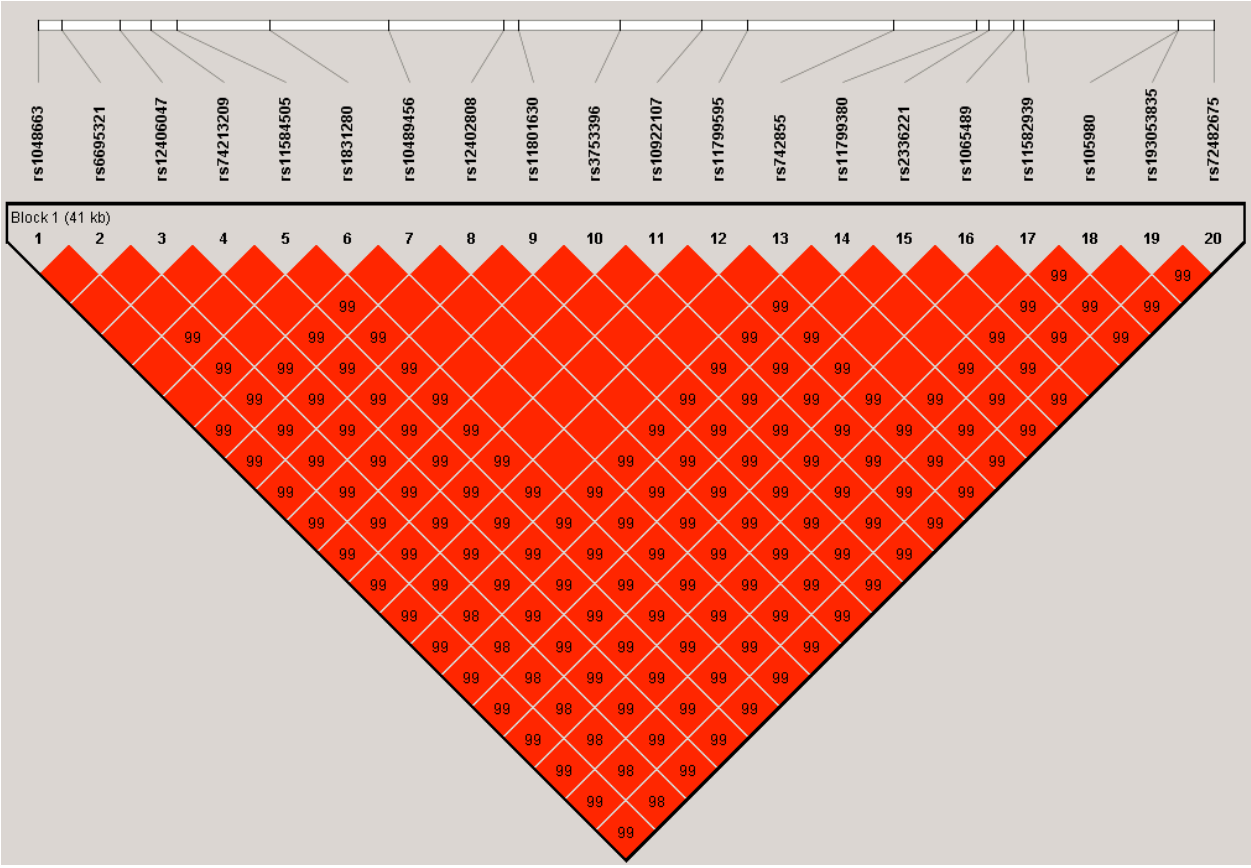
rs1048663
rs6695321
rs12406047
rs74213209
rs11584505
rs1831280
rs10489456
rs12402808
rs11801630
rs3753396
rs10922107
rs11799595
rs742855
rs11799380
rs2336221
rs1065489
rs11582939
rs105980
rs193053835
rs72482675
Block 1 (41 kb)
1 2 3 4 5 6 7 8 9 10 11 12 13 14 15 16 17 18 19 20

Figure S7C.

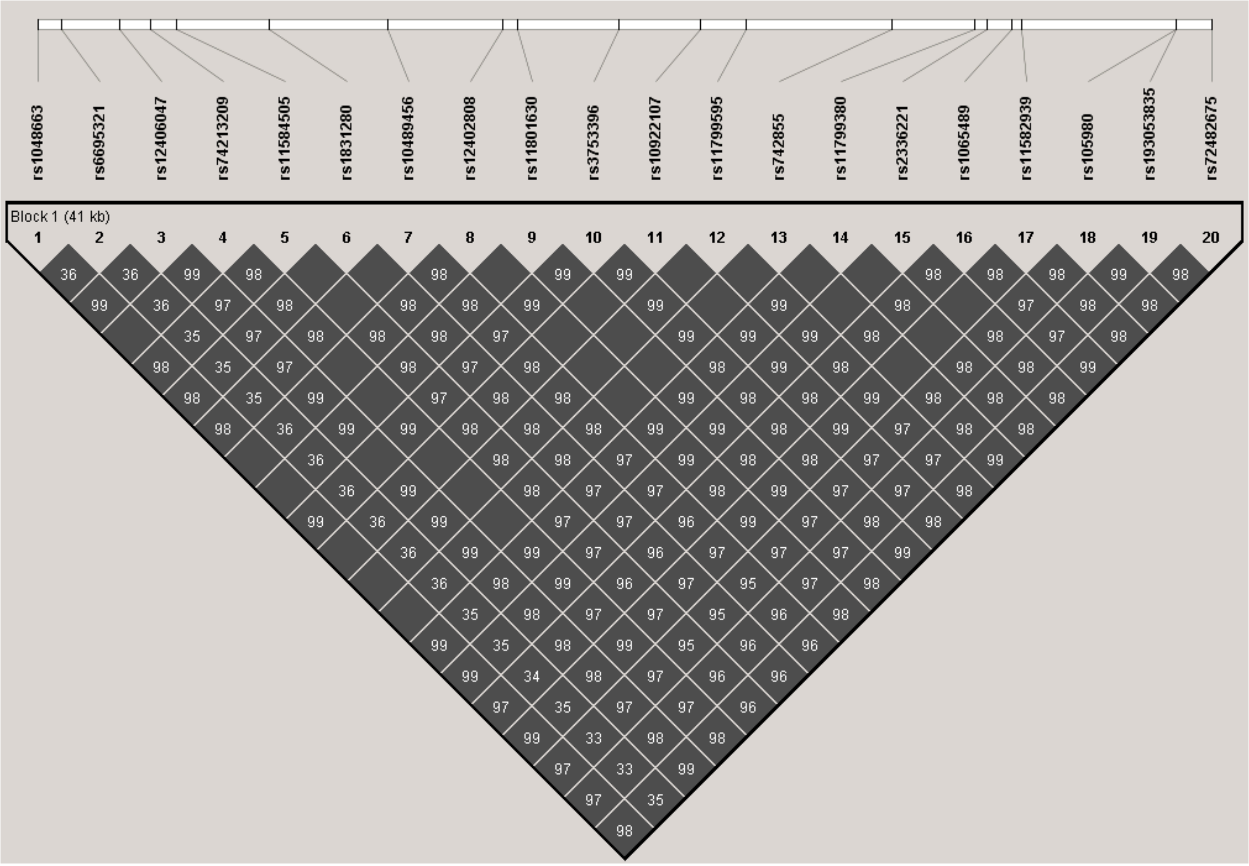
rs1048663
rs6695321
rs12406047
rs74213209
rs11584505
rs1831280
rs10489456
rs12402808
rs11801630
rs3753396
rs10922107
rs11799595
rs742855
rs11799380
rs2336221
rs1065489
rs11582939
rs105980
rs193053835
rs72482675
Block 1 (41 kb)
1 2 3 4 5 6 7 8 9 10 11 12 13 14 15 16 17 18 19 20

Figure S7D.

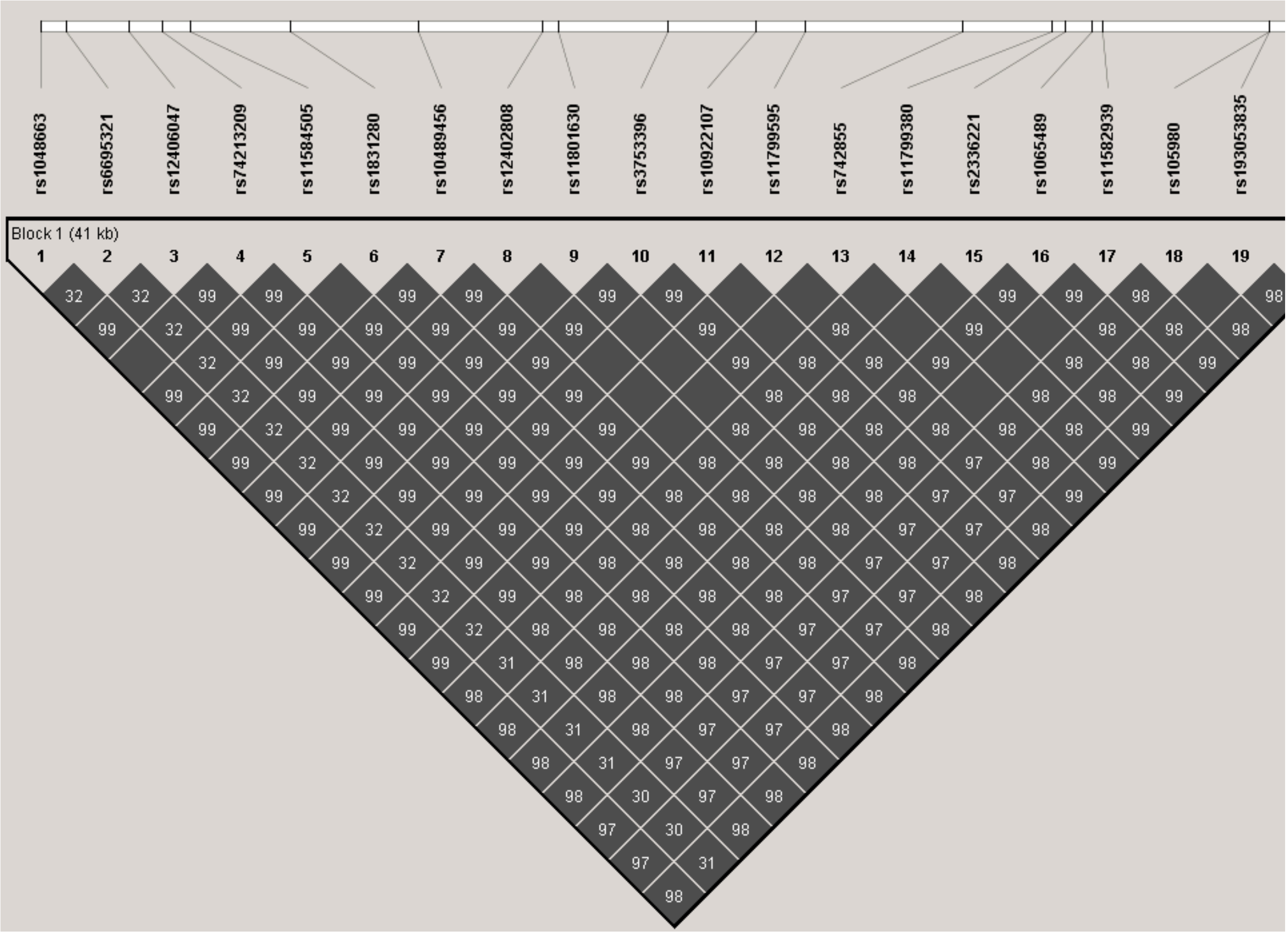
rs1048663
rs6695321
rs12406047
rs74213209
rs11584505
rs1831280
rs10489456
rs12402808
rs11801630
rs3753396
rs10922107
rs11799595
rs742855
rs11799380
rs2336221
rs1065489
rs11582939
rs105980
rs193053835
Block 1 (41 kb)
1 2 3 4 5 6 7 8 9 10 11 12 13 14 15 16 17 18 19

Figure S8A.

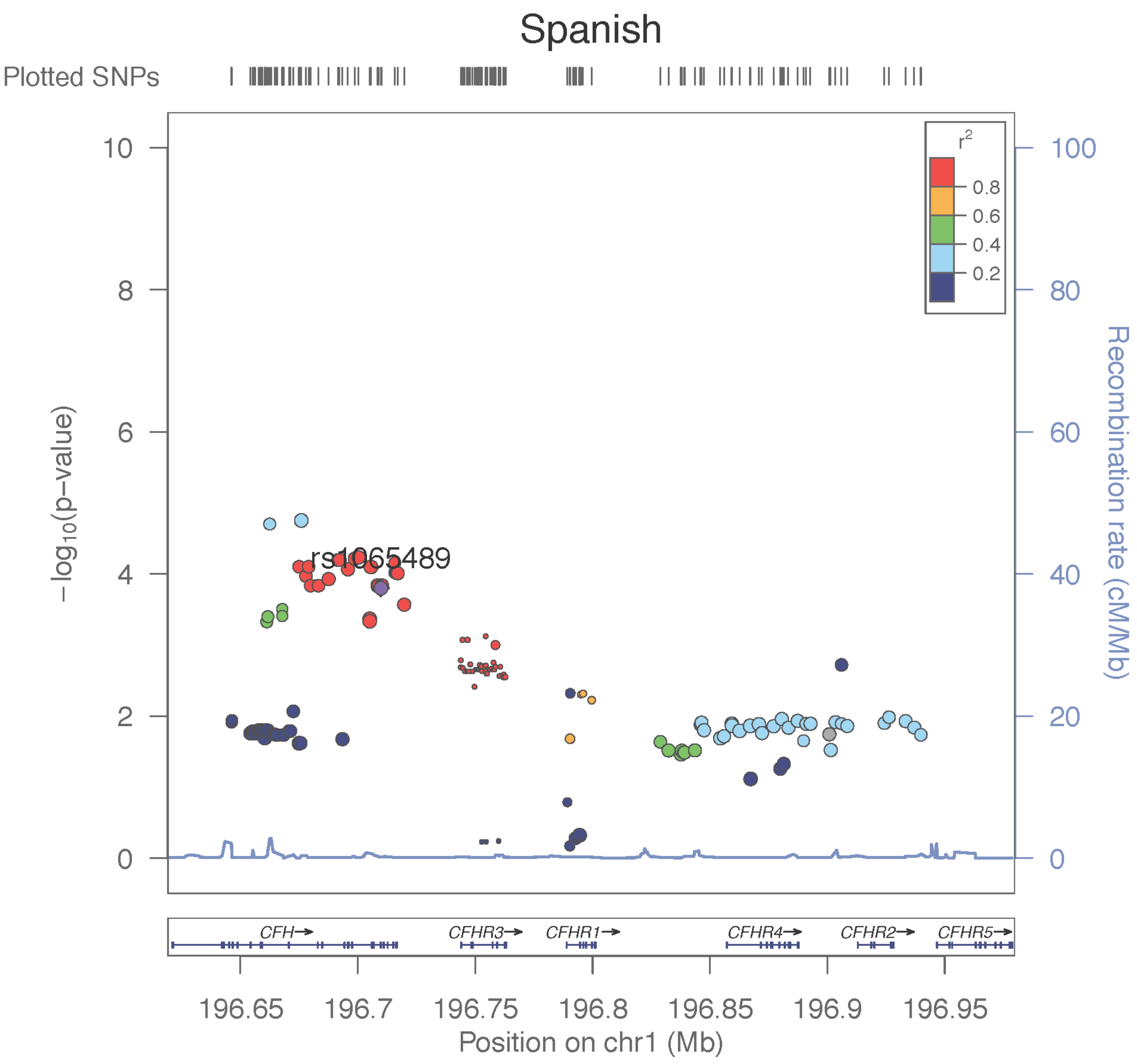
Spanish
Plotted SNPs
rs1065489
r2
0.8
0.6
0.4
0.2
−log10(p−value)
Recombination rate (cM/Mb)
CFH
CFHR3
CFHR1
CFHR4
CFHR2
CFHR5
196.65
196.7
196.75
196.8
196.85
196.9
196.95
Position on chr1 (Mb)

Figure S8B.

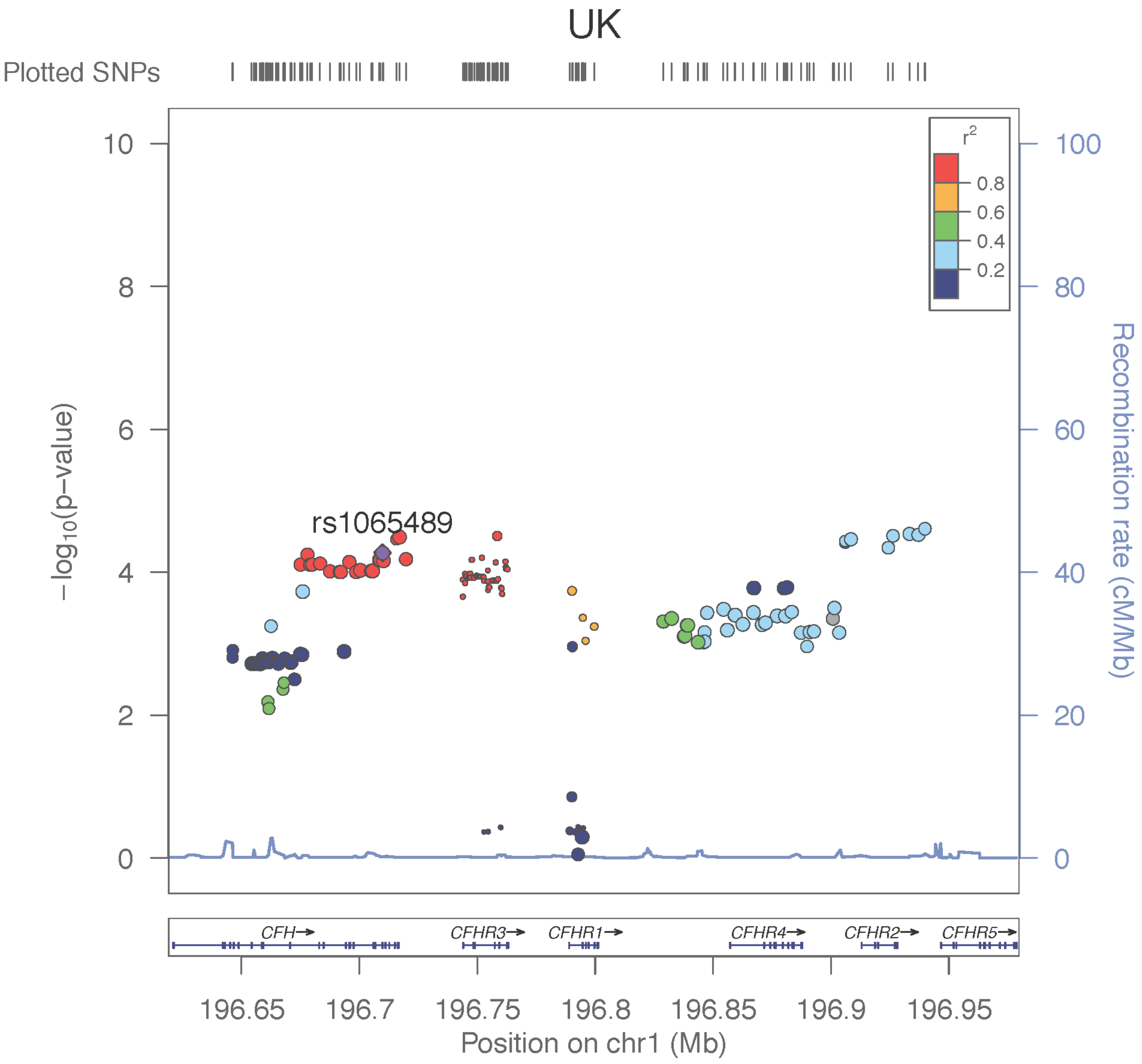
UK
Plotted SNPs
rs1065489
r2
0.8
0.6
0.4
0.2
10
8
6
4
2
0
100
80
60
40
20
0
−log10(p−value)
Recombination rate (cM/Mb)
CFH
CFHR3
CFHR1
CFHR4
CFHR2
CFHR5
196.65
196.7
196.75
196.8
196.85
196.9
196.95
Position on chr1 (Mb)

Figure S8C.

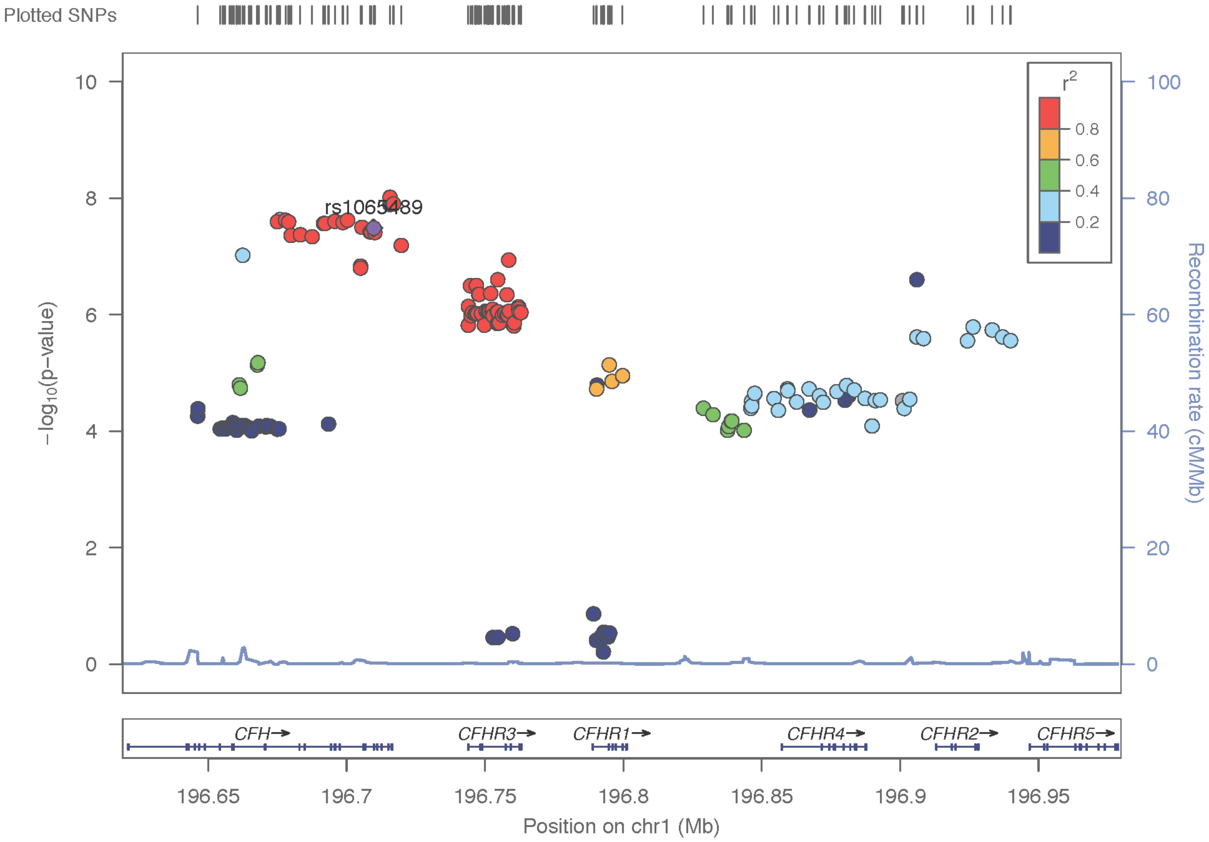

Plotted SNPs
rs1065489
r²
0.8
0.6
0.4
0.2
−log10(p−value)
Recombination rate (cM/Mb)
0
2
4
6
8
10
0
20
40
60
80
100
CFH→
CFHR3→
CFHR1→
CFHR4→
CFHR2→
CFHR5→
196.65
196.7
196.75
196.8
196.85
196.9
196.95
Position on chr1 (Mb)

Table S1. Population parameters of the candidate SNPs observed in the meta-analysis carried out in the present study using ENGINES.

Populations: AFRICA (*n* = 246), EUROPE (*n* = 380), EAST ASIA (*n* = 286), AMERICA (*n* = 181)

SNP	chromosome	position	genes	reference	ancestral	var	population	N	freq_A	freq_C	freq_G	freq_T	MA	MAF	Hobs	Hexp	Fs	Fst	In
rs72482675	chr01	196716924	CFH	C	C	CG	All	1093	0	0.768	0.232	0	G	0.232	0.296	0.357	-	0.156	0.081
rs72482675	chr01	196716924	CFH	C	C	CG	AFRICA	246	0	0.963	0.037	0	G	0.037	0.073	0.07	-	0.021	0.011
rs72482675	chr01	196716924	CFH	C	C	CG	EUROPE	380	0	0.817	0.183	0	G	0.183	0.292	0.299	-	0.016	0.027
rs72482675	chr01	196716924	CFH	C	C	CG	EAST ASIA	286	0	0.505	0.495	0	G	0.495	0.5	0.5	-	0.003	0.002
rs72482675	chr01	196716924	CFH	C	C	CG	AMERICA	181	0	0.812	0.188	0	G	0.188	0.287	0.305	-	0.03	0.016
rs105980	chr01	196715666	CFH	G	C	GA	All	1093	0.241	0	0.759	0	A	0.241	0.311	0.365	-	0.128	0.062
rs105980	chr01	196715666	CFH	G	C	GA	AFRICA	246	0.083	0	0.917	0	A	0.083	0.15	0.153	-	0.011	0.006
rs105980	chr01	196715666	CFH	G	C	GA	EUROPE	380	0.183	0	0.817	0	A	0.183	0.292	0.299	-	0.016	0.027
rs105980	chr01	196715666	CFH	G	C	GA	EAST ASIA	286	0.49	0	0.51	0	A	0.49	0.503	0.5	-	0.003	0.002
rs105980	chr01	196715666	CFH	G	C	GA	AMERICA	181	0.182	0	0.818	0	A	0.182	0.265	0.298	-	0.028	0.015
rs6695321	chr01	196675861	CFH	A	A	AG	All	1093	0.51	0	0.49	0	G	0.49	0.356	0.5	-	0.248	0.137
rs6695321	chr01	196675861	CFH	A	A	AG	AFRICA	246	0.833	0	0.167	0	G	0.167	0.276	0.278	-	0.019	0.01
rs6695321	chr01	196675861	CFH	A	A	AG	EUROPE	380	0.589	0	0.411	0	G	0.411	0.447	0.484	-	0.013	0.016
rs6695321	chr01	196675861	CFH	A	A	AG	EAST ASIA	286	0.138	0	0.862	0	A	0.138	0.255	0.238	-	0.015	0.007
rs6695321	chr01	196675861	CFH	A	A	AG	AMERICA	181	0.492	0	0.508	0	A	0.492	0.431	0.5	-	0.026	0.013
rs12406047	chr01	196677898	CFH	A	A	AT	All	1093	0.768	0	0	0.232	T	0.232	0.294	0.356	-	0.16	0.084
rs12406047	chr01	196677898	CFH	A	A	AT	AFRICA	246	0.965	0	0	0.035	T	0.035	0.069	0.067	-	0.023	0.012
rs12406047	chr01	196677898	CFH	A	A	AT	EUROPE	380	0.817	0	0	0.183	T	0.183	0.292	0.299	-	0.016	0.027
rs12406047	chr01	196677898	CFH	A	A	AT	EAST ASIA	286	0.502	0	0	0.498	T	0.498	0.5	0.5	-	0.004	0.002
rs12406047	chr01	196677898	CFH	A	A	AT	AMERICA	181	0.818	0	0	0.182	T	0.182	0.276	0.298	-	0.034	0.018
rs11799595	chr01	196700322	CFH	G	G	GC	All	1093	0	0.242	0.758	0	C	0.242	0.308	0.367	-	0.139	0.068
rs11799595	chr01	196700322	CFH	G	G	GC	AFRICA	246	0	0.073	0.927	0	C	0.073	0.138	0.136	-	0.017	0.009
rs11799595	chr01	196700322	CFH	G	G	GC	EUROPE	380	0	0.183	0.817	0	C	0.183	0.292	0.299	-	0.016	0.027
rs11799595	chr01	196700322	CFH	G	G	GC	EAST ASIA	286	0	0.5	0.5	0	C	0.5	0.497	0.5	-	0.003	0.002
rs11799595	chr01	196700322	CFH	G	G	GC	AMERICA	181	0	0.188	0.812	0	C	0.188	0.276	0.305	-	0.033	0.018
rs3753396	chr01	196695742	CFH	A	A	AG	All	1093	0.767	0	0.233	0	G	0.233	0.295	0.358	-	0.16	0.083
rs3753396	chr01	196695742	CFH	A	A	AG	AFRICA	246	0.963	0	0.037	0	G	0.037	0.073	0.07	-	0.021	0.011
rs3753396	chr01	196695742	CFH	A	A	AG	EUROPE	380	0.817	0	0.183	0	G	0.183	0.292	0.299	-	0.016	0.027
rs3753396	chr01	196695742	CFH	A	A	AG	EAST ASIA	286	0.5	0	0.5	0	A	0.5	0.497	0.5	-	0.003	0.002
rs3753396	chr01	196695742	CFH	A	A	AG	AMERICA	181	0.815	0	0.185	0	G	0.185	0.282	0.302	-	0.033	0.018
rs1048663	chr01	196674982	CFH	G	G	GA	All	1093	0.241	0	0.759	0	A	0.241	0.308	0.366	-	0.138	0.068
rs1048663	chr01	196674982	CFH	G	G	GA	AFRICA	246	0.073	0	0.927	0	A	0.073	0.138	0.136	-	0.021	0.011
rs1048663	chr01	196674982	CFH	G	G	GA	EUROPE	380	0.183	0	0.817	0	A	0.183	0.292	0.299	-	0.016	0.027
rs1048663	chr01	196674982	CFH	G	G	GA	EAST ASIA	286	0.498	0	0.502	0	A	0.498	0.5	0.5	-	0.004	0.002
rs1048663	chr01	196674982	CFH	G	G	GA	AMERICA	181	0.185	0	0.815	0	A	0.185	0.271	0.302	-	0.033	0.018
rs74213209	chr01	196679010	CFH	A	A	AG	All	1093	0.759	0	0.241	0	G	0.241	0.307	0.365	-	0.139	0.068
rs74213209	chr01	196679010	CFH	A	A	AG	AFRICA	246	0.929	0	0.071	0	G	0.071	0.134	0.132	-	0.018	0.01
rs74213209	chr01	196679010	CFH	A	A	AG	EUROPE	380	0.817	0	0.183	0	G	0.183	0.292	0.299	-	0.016	0.027
rs74213209	chr01	196679010	CFH	A	A	AG	EAST ASIA	286	0.502	0	0.498	0	G	0.498	0.5	0.5	-	0.004	0.002
rs74213209	chr01	196679010	CFH	A	A	AG	AMERICA	181	0.815	0	0.185	0	G	0.185	0.271	0.302	-	0.033	0.018
rs10922107	chr01	196698651	CFH	A	A	AT	All	1093	0.758	0	0	0.242	T	0.242	0.308	0.367	-	0.139	0.068
rs10922107	chr01	196698651	CFH	A	A	AT	AFRICA	246	0.927	0	0	0.073	T	0.073	0.138	0.136	-	0.017	0.009
rs10922107	chr01	196698651	CFH	A	A	AT	EUROPE	380	0.817	0	0	0.183	T	0.183	0.292	0.299	-	0.016	0.027
rs10922107	chr01	196698651	CFH	A	A	AT	EAST ASIA	286	0.5	0	0	0.5	A	0.5	0.497	0.5	-	0.003	0.002
rs10922107	chr01	196698651	CFH	A	A	AT	AMERICA	181	0.812	0	0	0.188	T	0.188	0.276	0.305	-	0.033	0.018
rs12402808	chr01	196691625	CFH	C	C	CA	All	1093	0.242	0.758	0	0	A	0.242	0.308	0.367	-	0.139	0.068
rs12402808	chr01	196691625	CFH	C	C	CA	AFRICA	246	0.073	0.927	0	0	A	0.073	0.138	0.136	-	0.017	0.009
rs12402808	chr01	196691625	CFH	C	C	CA	EUROPE	380	0.183	0.817	0	0	A	0.183	0.292	0.299	-	0.016	0.027
rs12402808	chr01	196691625	CFH	C	C	CA	EAST ASIA	286	0.5	0.5	0	0	A	0.5	0.497	0.5	-	0.003	0.002
rs12402808	chr01	196691625	CFH	C	C	CA	AMERICA	181	0.188	0.812	0	0	A	0.188	0.276	0.305	-	0.033	0.018
rs11801630	chr01	196692148	CFH	C	C	CT	All	1093	0	0.758	0	0.242	T	0.242	0.308	0.367	-	0.139	0.068
rs11801630	chr01	196692148	CFH	C	C	CT	AFRICA	246	0	0.927	0	0.073	T	0.073	0.138	0.136	-	0.017	0.009
rs11801630	chr01	196692148	CFH	C	C	CT	EUROPE	380	0	0.817	0	0.183	T	0.183	0.292	0.299	-	0.016	0.027
rs11801630	chr01	196692148	CFH	C	C	CT	EAST ASIA	286	0	0.5	0	0.5	C	0.5	0.497	0.5	-	0.003	0.002
rs11801630	chr01	196692148	CFH	C	C	CT	AMERICA	181	0	0.812	0	0.188	T	0.188	0.276	0.305	-	0.033	0.018
rs742855	chr01	196705520	CFH	A	A	TC	All	1093	0	0.242	0	0.758	C	0.242	0.309	0.367	-	0.138	0.067
rs742855	chr01	196705520	CFH	A	A	TC	AFRICA	246	0	0.075	0	0.925	C	0.075	0.142	0.139	-	0.016	0.009
rs742855	chr01	196705520	CFH	A	A	TC	EUROPE	380	0	0.183	0	0.817	C	0.183	0.292	0.299	-	0.016	0.027
rs742855	chr01	196705520	CFH	A	A	TC	EAST ASIA	286	0	0.5	0	0.5	C	0.5	0.497	0.5	-	0.003	0.002
rs742855	chr01	196705520	CFH	A	A	TC	AMERICA	181	0	0.188	0	0.812	C	0.188	0.276	0.305	-	0.033	0.018
rs1065489	chr01	196709774	CFH	G	G	GT	All	1093	0	0	0.767	0.233	T	0.233	0.298	0.358	-	0.157	0.082
rs1065489	chr01	196709774	CFH	G	G	GT	AFRICA	246	0	0	0.963	0.037	T	0.037	0.073	0.07	-	0.021	0.011
rs1065489	chr01	196709774	CFH	G	G	GT	EUROPE	380	0	0	0.817	0.183	T	0.183	0.292	0.299	-	0.016	0.027
rs1065489	chr01	196709774	CFH	G	G	GT	EAST ASIA	286	0	0	0.503	0.497	T	0.497	0.503	0.5	-	0.002	0.001
rs1065489	chr01	196709774	CFH	G	G	GT	AMERICA	181	0	0	0.809	0.191	T	0.191	0.293	0.309	-	0.032	0.017
rs11799380	chr01	196708455	CFH	A	A	AG	All	1093	0.758	0	0.242	0	G	0.242	0.309	0.366	-	0.136	0.067
rs11799380	chr01	196708455	CFH	A	A	AG	AFRICA	246	0.927	0	0.073	0	G	0.073	0.138	0.136	-	0.017	0.009
rs11799380	chr01	196708455	CFH	A	A	AG	EUROPE	380	0.817	0	0.183	0	G	0.183	0.292	0.299	-	0.016	0.027
rs11799380	chr01	196708455	CFH	A	A	AG	EAST ASIA	286	0.503	0	0.497	0	G	0.497	0.497	0.5	-	0.002	0.001
rs11799380	chr01	196708455	CFH	A	A	AG	AMERICA	181	0.809	0	0.191	0	G	0.191	0.282	0.309	-	0.029	0.016
rs2336221	chr01	196708891	CFH	G	G	GT	All	1093	0	0	0.758	0.242	T	0.242	0.309	0.366	-	0.136	0.067
rs2336221	chr01	196708891	CFH	G	G	GT	AFRICA	246	0	0	0.927	0.073	T	0.073	0.138	0.136	-	0.017	0.009
rs2336221	chr01	196708891	CFH	G	G	GT	EUROPE	380	0	0	0.817	0.183	T	0.183	0.292	0.299	-	0.016	0.027
rs2336221	chr01	196708891	CFH	G	G	GT	EAST ASIA	286	0	0	0.503	0.497	T	0.497	0.497	0.5	-	0.002	0.001
rs2336221	chr01	196708891	CFH	G	G	GT	AMERICA	181	0	0	0.809	0.191	T	0.191	0.282	0.309	-	0.029	0.016
rs11582939	chr01	196710157	CFH	C	C	CT	All	1093	0	0.759	0	0.241	T	0.241	0.31	0.366	-	0.135	0.066
rs11582939	chr01	196710157	CFH	C	C	CT	AFRICA	246	0	0.927	0	0.073	T	0.073	0.138	0.136	-	0.017	0.009
rs11582939	chr01	196710157	CFH	C	C	CT	EUROPE	380	0	0.817	0	0.183	T	0.183	0.292	0.299	-	0.016	0.027
rs11582939	chr01	196710157	CFH	C	C	CT	EAST ASIA	286	0	0.505	0	0.495	T	0.495	0.5	0.5	-	0.003	0.002
rs11582939	chr01	196710157	CFH	C	C	CT	AMERICA	181	0	0.809	0	0.191	T	0.191	0.282	0.309	-	0.029	0.016
rs1831280	chr01	196683274	CFH	T	T	AG	All	1093	0.754	0	0.246	0	G	0.246	0.316	0.371	-	0.134	0.065
rs1831280	chr01	196683274	CFH	T	T	AG	AFRICA	246	0.917	0	0.083	0	G	0.083	0.159	0.153	-	0.014	0.008
rs1831280	chr01	196683274	CFH	T	T	AG	EUROPE	380	0.814	0	0.186	0	G	0.186	0.297	0.302	-	0.016	0.027
rs1831280	chr01	196683274	CFH	T	T	AG	EAST ASIA	286	0.498	0	0.502	0	A	0.498	0.5	0.5	-	0.003	0.001
rs1831280	chr01	196683274	CFH	T	T	AG	AMERICA	181	0.812	0	0.188	0	G	0.188	0.276	0.305	-	0.033	0.018
rs11584505	chr01	196679927	CFH	A	A	AC	All	1093	0.755	0.245	0	0	C	0.245	0.315	0.37	-	0.133	0.064
rs11584505	chr01	196679927	CFH	A	A	AC	AFRICA	246	0.917	0.083	0	0	C	0.083	0.159	0.153	-	0.014	0.008
rs11584505	chr01	196679927	CFH	A	A	AC	EUROPE	380	0.814	0.186	0	0	C	0.186	0.297	0.302	-	0.016	0.027
rs11584505	chr01	196679927	CFH	A	A	AC	EAST ASIA	286	0.5	0.5	0	0	A	0.5	0.497	0.5	-	0.003	0.002
rs11584505	chr01	196679927	CFH	A	A	AC	AMERICA	181	0.812	0.188	0	0	C	0.188	0.276	0.305	-	0.033	0.018
rs10489456	chr01	196687515	CFH	C	C	GA	All	1093	0.246	0	0.754	0	A	0.246	0.317	0.371	-	0.131	0.063
rs10489456	chr01	196687515	CFH	C	C	GA	AFRICA	246	0.087	0	0.913	0	A	0.087	0.167	0.16	-	0.014	0.008
rs10489456	chr01	196687515	CFH	C	C	GA	EUROPE	380	0.186	0	0.814	0	A	0.186	0.297	0.302	-	0.016	0.027
rs10489456	chr01	196687515	CFH	C	C	GA	EAST ASIA	286	0.5	0	0.5	0	A	0.5	0.497	0.5	-	0.003	0.002
rs10489456	chr01	196687515	CFH	C	C	GA	AMERICA	181	0.188	0	0.812	0	A	0.188	0.276	0.305	-	0.033	0.018
rs529541	chr01	196719716	-	T	C	AG	All	1093	0.697	0	0.303	0	G	0.303	0.361	0.423	-	0.084	0.039

rs529541	chr01	196719716	-	T	C	AG	AFRICA	246	0.671	0	0.329	0	G	0.329	0.382	0.442	-	0.028	0.013
rs529541	chr01	196719716	-	T	C	AG	EUROPE	380	0.817	0	0.183	0	G	0.183	0.292	0.299	-	0.016	0.027
rs529541	chr01	196719716	-	T	C	AG	EAST ASIA	286	0.493	0	0.507	0	A	0.493	0.476	0.5	-	0.004	0.002
rs529541	chr01	196719716	-	T	C	AG	AMERICA	181	0.801	0	0.199	0	G	0.199	0.298	0.319	-	0.034	0.018
rs10922096	chr01	196662459	CFH	T	T	TC	All	1093	0	0.526	0	0.474	T	0.474	0.355	0.499	-	0.249	0.143
rs10922096	chr01	196662459	CFH	T	T	TC	AFRICA	246	0	0.152	0	0.848	C	0.152	0.248	0.258	-	0.031	0.016
rs10922096	chr01	196662459	CFH	T	T	TC	EUROPE	380	0	0.489	0	0.511	C	0.489	0.458	0.5	-	0.011	0.019
rs10922096	chr01	196662459	CFH	T	T	TC	EAST ASIA	286	0	0.864	0	0.136	T	0.136	0.252	0.236	-	0.013	0.007
rs10922096	chr01	196662459	CFH	T	T	TC	AMERICA	181	0	0.577	0	0.423	T	0.423	0.448	0.488	-	0.02	0.01
rs70620	chr01	196704997	CFH	C	T	GA	All	1093	0.313	0	0.687	0	A	0.313	0.39	0.43	-	0.082	0.038
rs70620	chr01	196704997	CFH	C	T	GA	AFRICA	246	0.372	0	0.628	0	A	0.372	0.467	0.467	-	0.018	0.008
rs70620	chr01	196704997	CFH	C	T	GA	EUROPE	380	0.183	0	0.817	0	A	0.183	0.292	0.299	-	0.016	0.027
rs70620	chr01	196704997	CFH	C	T	GA	EAST ASIA	286	0.5	0	0.5	0	A	0.5	0.497	0.5	-	0.003	0.002
rs70620	chr01	196704997	CFH	C	T	GA	AMERICA	181	0.21	0	0.79	0	A	0.21	0.32	0.332	-	0.029	0.015
rs70621	chr01	196705009	CFH	C	C	GA	All	1093	0.29	0	0.71	0	A	0.29	0.375	0.412	-	0.082	0.038
rs70621	chr01	196705009	CFH	C	C	GA	AFRICA	246	0.28	0	0.72	0	A	0.28	0.423	0.404	-	0.034	0.017
rs70621	chr01	196705009	CFH	C	C	GA	EUROPE	380	0.183	0	0.817	0	A	0.183	0.292	0.299	-	0.016	0.027
rs70621	chr01	196705009	CFH	C	C	GA	EAST ASIA	286	0.5	0	0.5	0	A	0.5	0.497	0.5	-	0.003	0.002
rs70621	chr01	196705009	CFH	C	C	GA	AMERICA	181	0.196	0	0.804	0	A	0.196	0.293	0.315	-	0.038	0.021
rs28434450	chr02	224593929	-	T	T	TG	All	1093	0	0	0.323	0.677	G	0.323	0.431	0.437	-	0.008	0.003
rs28434450	chr02	224593929	-	T	T	TG	AFRICA	246	0	0	0.337	0.663	G	0.337	0.423	0.447	-	0.005	0.002
rs28434450	chr02	224593929	-	T	T	TG	EUROPE	380	0	0	0.267	0.733	G	0.267	0.397	0.392	-	0.004	0.002
rs28434450	chr02	224593929	-	T	T	TG	EAST ASIA	286	0	0	0.365	0.635	G	0.365	0.451	0.464	-	0.008	0.004
rs28434450	chr02	224593929	-	T	T	TG	AMERICA	181	0	0	0.351	0.649	G	0.351	0.481	0.455	-	0.013	0.007

Table S2. Pathogenicity and deleteriousnes scores at rs106548[illegible]

rs1065489	#
**#Chr**	1
**Start**	196709774
**End**	196709774
**Ref**	G
**Alt**	T
**SIFT_score**	0.593
**SIFT_pred**	T
**Polyphen2_HDIV_score**	0.75
**Polyphen2_HDIV_pred**	P
**Polyphen2_HVAR_score**	0.196
**Polyphen2_HVAR_pred**	B
**LRT_score**	.
**LRT_pred**	.
**MutationTaster_score**	1
**MutationTaster_pred**	P
**MutationAssessor_score**	0.62
**MutationAssessor_pred**	N
**FATHMM_score**	-0.02
**FATHMM_pred**	T
**PROVEAN_score**	-0.89
**PROVEAN_pred**	N
**VEST3_score**	0.07
**CADD_raw**	-0.535
**CADD_phred**	0.18
**DANN_score**	0.985
**fathmm-MKL_coding_score**	0.032
**fathmm-MKL_coding_pred**	N
**MetaSVM_score**	-0.954
**MetaSVM_pred**	T
**MetaLR_score**	0
**MetaLR_pred**	T
**integrated_fitCons_score**	0.706
**integrated_confidence_value**	0
**GERP++_RS**	-2.39
**phyloP7way_vertebrate**	-0.31
**phyloP20way_mammalian**	-0.304
**phastCons7way_vertebrate**	0.001
**phastCons20way_mammalianSiPhy_29way_l[illegible]**	0.002
